# Key stakeholder experiences of an integrated healthcare pilot in Australia: a thematic analysis

**DOI:** 10.1186/s12913-020-05794-2

**Published:** 2020-10-07

**Authors:** Steven A. Trankle, Tim Usherwood, Penelope Abbott, Mary Roberts, Michael Crampton, Christian M. Girgis, John Riskallah, Yashu Chang, Jaspreet Saini, Jennifer Reath

**Affiliations:** 1 Department General Practice, School of Medicine, Western Sydney University, Building 30.3.18 Campbelltown Campus, Locked Bag 1797, Penrith, NSW 2751 Australia; 2 Westmead Clinical School, Sydney Medical School, The University of Sydney, Sydney, Australia; 3 Western Sydney Local Health District (Westmead Hospital), Sydney, Australia; 4 George Institute for Global Health, Sydney, Australia; 5 Western Sydney Primary Health Network, Sydney, Australia; 6 Royal North Shore Hospital, Sydney, Australia; 7 Western Sydney Local Health District (Blacktown Hospital), Sydney, Australia

**Keywords:** Integrated care, Chronic illness, Qualitative, Australia, General practice

## Abstract

**Background:**

In Australia and other developed countries, chronic illness prevalence is increasing, as are costs of healthcare, particularly hospital-based care. Integrating healthcare and supporting illness management in the community can be a means of preventing illness, improving outcomes and reducing unnecessary hospitalisation. Western Sydney has high rates of diabetes, heart and respiratory diseases and the NSW State Ministry of Health funded a range of key strategies through the Western Sydney Integrated Care Program (WSICP) to integrate care across hospital and community settings for patients with these illnesses. Complementing our previously reported analysis related to specific WSICP strategies, this research provided information concerning overall experiences and perspectives of WSICP implementation and integrated care generally.

**Methods:**

We administered 125 in-depth interviews in two rounds over 12 months with 83 participants including patients and their carers, care facilitators, hospital specialists and nurses, allied health professionals, general practitioners and primary care nurses, and program managers. Half of the participants (*n* = 42) were interviewed twice. We conducted an inductive, thematic analysis on the interview transcripts.

**Results:**

Key themes related to the set-up and operationalising of WSICP; challenges encountered; and the added value of the program. Implementing WSICP was a large and time consuming undertaking but challenges including those with staffing and information technology were being addressed. The WSICP was considered valuable in reducing hospital admissions due to improved patient self-management and a focus on prevention, greater communication and collaboration between healthcare providers across health sectors and an increased capacity to manage chronic illness in the primary care setting.

**Conclusions:**

Patients, carers and health providers experienced the WSICP as an innovative integrated care model and valued its patient-centred approach which was perceived to improve access to care, increase patient self-management and illness prevention, and reduce hospital admissions. Long-term sustainability of the WSICP will depend on retaining key staff, more effectively sharing information including across health sectors to support enhanced collaboration, and expanding the suite of activities into other illness areas and locations. Enhanced support for general practices to manage chronic illness in the community, in collaboration with hospital specialists is critical. Timely evaluation informs ongoing program implementation.

## Background

Integrated healthcare is advocated internationally as a means of addressing the increasing prevalence of chronic illness and the need to deliver quality care within restricted budgets [1, 2]. The evidence suggests that service users benefit when care is coordinated around the needs of people and their communities by organisations and services working together [3, 4]. Integrated health systems improve access, quality and continuity of services, especially for people with complex needs and multiple morbidity [2, 5, 6], by linking primary and secondary care and addressing allied health and social needs [3, 7]. An integrated health system relies on trust between different service providers, common understandings of integrated care and shared goals [8–12]. Strong primary care and a consumer focus are key features of integrated healthcare programs for chronic illnesses [6, 13].

Chronic illness is major cause of hospitalisation in Australia, yet most admissions are for deteriorating conditions that could be prevented or managed in the community [14]. In Australia, Australian government funded Primary Health Networks support primary health care and general practices, whilst State government funded Local Health Districts manage public health care in hospital settings, as well as some community health and preventive health services. Primary health care in Australia is funded through a fee-for-service model which is not as well-suited to managing chronic illness as a bundled or blended form of payment [15, 16].

The western Sydney region has high rates of diabetes, cardiac failure and respiratory diseases in comparison to other parts of Australia [17, 18]. To address these health issues, the Western Sydney Local Health District (WSLHD) and the Western Sydney Primary Health Network (WSPHN) worked in partnership to develop and implement the Western Sydney Integrated Care Program (WSICP) Demonstrator. Run as a pilot from 2014 to 2017 [19], the WSICP was aligned with Bodenheimer and Sinsky’s quadruple aim [20]:

1. improve the health of patients with chronic illness;
2. enhance patient experience;
3. reduce costs of healthcare; and
4. better support health professionals in caring for these patients.

Informed by the current evidence for integrating healthcare [3, 21–23], the WSICP implemented a range of strategies [19] (Table 1), targeted to patients with diabetes, congestive cardiac failure (CCF) and chronic obstructive pulmonary disease (COPD) [17, 24].

**Table 1:** WSICP Initiatives

WSICP Strategies	Definition
**Care Facilitators**	Employed by the WSLHD to help support and coordinate patient services, and link patients, general practice, hospital and other service providers
**Information Technology (IT)**	Initiatives to establish a shared health record, to enhance communication between hospital and community sectors
**Shared Care Plans**	Created in general practices for sharing with hospitals, community health providers and patients through the “Linked Electronic Health Record (EHR)” (above)
**Specialist Action Plans**	Provided at hospital discharge to inform patients and general practitioners about care in complex and frequently changing situations
**GP Support Line**	For GPs to access hospital specialists faster and refer patients to rapid access clinics as needed
**Rapid Access and Stabilisation Service (RASS) Clinics**	For reducing unnecessary hospital admission and re-admission and includes Patient Hotlines to improve patient access to the clinics
**HealthPathways**	GP access to online guidelines and local referral information
**Support Payments to General Practices**	To facilitate patient enrolment and care planning
**Promotion of Patient Centred Medical Home**	Support for general practices to lead multidisciplinary teams that provide comprehensive coordinated care. Training and support provided to improve efficiency and use of information technology.
**Communication between WSICP and non-WSICP Services**	Building connections between hospital and other government and non-government services for patient needs.

Despite integrated care programs becoming more common, many do not achieve their expected results. Programs frequently fail at the implementation stage, and many do not plan early evaluation that informs their implementation [25].

### Research aims

We conducted two different qualitative approaches to evaluate the WSICP using a framework analysis and a thematic analysis. The framework analysis reported elsewhere [26] focused on the specific initiatives in the WSICP [27]. As we began coding data for this analysis, we noted a large amount of data related to the experiences and implementation of integrated care more generally. It was agreed that this data was valuable to explore and further inform the implementation of the WSICP. This paper describes a thematic analysis of this data. Using an inductive approach, we aimed to explore how different stakeholders experienced the WSICP and investigate their broader perspectives and experiences of integrated care.

## Methods

The methods used in the current analysis, particularly the study design, recruitment of participants and data collection are the same as used in our framework analysis of the WSICP [27].

### Design of the study

We conducted a qualitative evaluation [28, 29] through individual interviews across two rounds over a 12 month period between 2016 and 2017 to collect information on WSICP experiences including how this changed over time [30]. Initial interviews were conducted as early in the program as possible, allowing for sufficient patients to be enrolled. Follow up interviews were conducted on average eight-nine months later once participants had experience of the program. As a Demonstrator or pilot program, this timeframe enabled program managers to use this information to refine the ongoing roll out of the program. We used the COREQ criteria as a guide for reporting our research [31].

### The researchers and WSICP evaluation management

The primary research team from Western Sydney University (WSU) and University of Sydney included four experienced qualitative researchers working in the area of health services. We collaborated with western Sydney clinicians from the WSLHD and WSPHN, as co-investigators, who assisted us with recruitment and data interpretation. Three of the co-investigators were engaged in WSICP management. However, only one of these co-investigators assisted with data coding. We regularly reported progress to the Integrated Care Evaluation Advisory Committee which included clinical and administrative leaders who oversaw implementation of WSICP and its evaluation.

### Sampling and recruitment

In consultation with the Integrated Care Evaluation Advisory Committee, we developed a stratified sampling frame to collect the perspectives of all stakeholder groups involved in the WSICP. These included patients and carers, management staff-some with clinical roles, a range of clinical and allied healthcare providers from Westmead and Blacktown hospitals, and participating general practitioners (GPs) and general practice staff from western Sydney.

Patients and carers were recruited during their hospital appointments by the co-investigators and in the community by care facilitators. Hospital clinicians engaged in the WSICP were purposively sampled by WSICP program managers and clinical co-investigators. All participants received information on the study at the time of recruitment and the contact details for consenting participants were sent to the research manager (ST). Management staff were invited to participate by the researchers. General practitioners and their nursing staff were recruited by WSPHN staff and these participants mailed their contact details to ST after receiving information about the study. All participants provided written informed consent prior to their interview. Most participants did not know the research manager prior to the study, although other members of the research team were known to the GPs and hospital specialists.

We suspended recruitment to the first round of interviews as we approached our target of 70 participants. This sample ensured all stakeholder groups were adequately represented. Participants signed a pre-consent form for a second interview. We recruited patients and carers and staff from general practices in the second round using the same process as the first round. None of the referred participants declined participation, but some were not available for a second interview.

### Interviews (data collection)

In consultation with the Integrated Care Evaluation Advisory Committee, and informed by the literature, we designed a semi – structured interview guide to collect participants’ experiences and perspectives on the WSICP compared with their previous care experience. The university researchers refined the guide in meetings with program managers and clinical co-investigators. We used the first 10 interviews to pilot the guide and reviewed those transcripts to ensure the questions were clear and comprehensive. Through the course of the interviews we refined the guide across all participant groups by adding occasional questions and prompts to explore emerging areas of interest in more depth and withdrew a small number of questions that were not generating new information. This process is accepted practice with semi-structured interviews [32]. We further refined the interview guide before commencing the second round of interviews. This allowed us to investigate important issues arising from the first round as well as changes over time (Additional file 1).

Interviews were conducted one-on-one by a single interviewer (ST), mostly face to face in hospital and general practice offices although, where participants preferred this, some (40%) were conducted by telephone. Interviews took between 30 and 60 min, were audio-recorded and then transcribed by an independent transcription service. Interviewees were offered the opportunity to review their transcript after these had been checked for accuracy. Transcriptions were de-identified before being provided to the research team.

### Analysis

We conducted a thematic analysis using N-Vivo 11® software to aid organisation of data. Thematic analysis allows patterns and meanings to be captured from qualitative datasets which are important to the understanding of the research questions [33, 34]. Our thematic analysis was inductive and data-driven.

Initial coding of the first 10 interview transcripts was conducted by four research team members (ST, JR, TU, PA) and one clinical co-investigator (MR) who each coded up to three transcripts, with five additional transcripts cross-coded by two or more researchers to ensure consistency. This was an iterative process where transcripts were read a number of times to allow emerging aspects of interest to be captured.

The five researchers then met with the full research team to discuss the analysis and agreed on core initial themes for further ongoing analysis. ST then conducted the remaining interviews and continued to code all remaining transcripts. We met regularly with the wider research team to check and refine the emerging analysis. This was an ongoing process over a number of meetings as additional interview transcripts often revealed further areas of interest. The second round of interview transcripts was similarly managed. Additional codes were identified including from those who were interviewed twice. Upon achieving saturation of codes, agreement was reached on a final thematic structure which clearly and comprehensively described our analysis (Table 2).

**Table 2:** Thematic structure

Key Theme	Subtheme
**Setting up of WSICP**	• Initiation and promotion of the program
	• Access to WSICP
	• Understanding integrated care
	• Relationships with other unrelated programs, activities and processes
**Challenges**	• Interorganisational challenges
	• Challenges with roles and responsibilities
	• Scale of the undertaking
**Added Value of Integrated Care**	• Building capacity, education and upskilling
	• Changes in practice
	• Valuing WSICP
	• Suggestions

We reviewed our analysis with the Evaluation Advisory Committee of the WSICP at the end of each of the two interview rounds in order to enhance the trustworthiness of the findings. Although the Evaluation Advisory Committee was not involved in coding or theming, they confirmed our analysis in light of their experience of the program and reflected on how findings could inform ongoing delivery of WSICP. We took their comments back to the Research Team to consider in our interpretation and resolved any differences through a process of consensus.

## Results

We conducted 125 interviews with a total of 83 participants in two rounds between March and September 2016, and from November 2016 to March 2017 (Table 3). There were 59 interviewees in the first and 66 in the second round. These included 12 WSICP enrolled patients, seven of whom were interviewed in both rounds, and 11 carers. Only one carer was interviewed in both rounds due to difficulties recruiting these participants in the first round. We interviewed 29 healthcare providers from WSLHD including medical specialists, registrars, nurses, allied healthcare providers and WSICP care facilitators, and most of these (*n* = 20) participated in both rounds. We also interviewed 21 GPs and practice staff from different practices across western Sydney with eight of them interviewed twice. Ten managers and clinicians on the Evaluation Advisory Committee participated, with seven participating in both interview rounds. We sampled participants across the three chronic disease areas and in the catchment areas of both Westmead and Blacktown hospitals.

**Table 3:** Participants

Participant Type		First Round	Second Round	Total Participants^a^
Patients/carers	*Patients*	11	9	12
	*Carers*	1	10	11
	**Total Patients and Carers**	**12**	**19**	**23**
Healthcare providers	*Hospital specialists and registrars*	12	8	12
	*Hospital Nursing staff*	7	6	7
	*Hospital Allied Healthcare Providers*	5	4	6
	*General Practitioners (GPs)*	7	12	14
	*General Practice Nurses*	3	7	7
	*Care Facilitators*	3	3	4
	**Total Healthcare providers**	**37**	**40**	**50**
Evaluation Advisory Committee	*Entitled “Managers” in our results*	10	7	**10**
Total Participants		**59**	**66**	**83**

^a^Without duplications across rounds 1 and 2

### Thematic analysis

We identified three overarching themes in our thematic analysis. These related to the set-up and operationalising of WSICP; challenges encountered; and the added value of the program. The full analysis table is available as “Additional file 2”.

#### Setting up of WSICP

Interviews highlighted a range of subthemes related to managing the program in the early stages; initiation and promotion of the program; access to WSICP; understandings of integrated care; and relationships with other unrelated programs, activities and processes.

The first round of interviews highlighted the effort and time involved in setting up WSICP with lengthy delays perceived to be related to WSLHD bureaucracy. Hospital clinicians commented: *“That’s been quite stressful …it’s a lot of hours put in of our own time and private time too to get this up and running (Hospital Specialist 11, Round 1),* and *“…hospital processes held up the Integrated Care Program a lot…has been frustrating…that has slowed things down a lot” (Hospital Specialist 1/Manager 7, Round 1).* There were also delays initially in engaging GPs: *“it’s not having enough GPs at the start that have been enrolled in the actual program, so we were getting many of our referrals from inpatients” (Hospital Specialist 5, Round 1).*

Understanding of policies and processes as well as provision of staff orientation appeared to improve over time. Compared to care facilitators recruited early in the program, later care facilitators received full orientation and mentoring: *“New care facilitators coming on board have a different route to orientation to the way I was brought in, quite more substantial orientation than I received 12 months ago” (Care Facilitator 2, Round 2).*

Concerns about restrictions to WSICP access continued over time, with many who were perceived as likely to benefit excluded: *“I’ve got patients who have diabetes and a heart problem as well but the patient was not included, because the patient had non-Hodgkin’s lymphoma” (GP 13, Round 2)*. Access even for those who were eligible for WSICP was also a concern and patients were reported as missing appointments for reasons including illness, disability, limited English proficiency and financial barriers: *“Some of them I know feel that they’re too sick to come, some of them it’s too much effort to get back to the hospital, some of them forget, some of them misplace the timing, and [difficulties accessing] interpreters-ugh (Hospital Specialist 2/Manager 9, Round 1).* Poor physical access to hospital clinics and inadequate parking were also described as barriers: *“They miss their rehab sessions, miss therapy appointments, they ring and say, ‘I drove around for an hour and couldn’t get a car park, that’s why I didn’t come to my appointment today’” (Allied Health 5, Round 1).*

Rapid Access and Stabilisation clinics addressed some of these difficulties by adapting care to patient needs: *“We’ve changed to walk-in appointments to try and get them in with the 24–48 h, even five-day time frame (Hospital Nurse 2, Round 1).* Patients felt valued and important:

*…rapid access was magic, it was gold, treated like special rather than waiting in emergency for hours and hours feeling unwell, here I was being seen by people who met me at the door with a wheelchair and took me places to assess me (Patient 2, Round 1)*

However, it was difficult engaging patients who seemed disinterested in an integrated, team based approach: *“…having extra things to do or more people involved was actually a barrier. He wasn’t interested in signing up because the last thing he wanted was more phone calls or more appointments” (GP 6, Round 2).*

Patients, carers and providers demonstrated a good understanding of integrated care describing this in terms of a focus on patient centred care that was integrated across hospital, specialist, GP and community settings: *“In a nutshell I believe what integrated care is about - integrating three different groups of people, which is hospital, GP, the patient carer or a different family member” (Carer 17, Round 2).* Informational continuity was often part of their description with care facilitation and team based approaches frequently considered to be aspects of integrated care. Comments included: *“there’s continuity…healthcare plans are uploaded so other health professionals involved in their care can have an idea of what’s going on with what other people are doing (GP 12, Round 2),* and *“Shared-care, basically. So, we’re looking at a group of people to look after the one patient” (Practice Nurse 5, Round 2).*

The need for all to work together for the benefit of the patient was identified as paramount. Typical responses included: *“Somebody communicating with all the various specialities that take care of a patient. So, there’s one person overlooking it all and making sure everything is working well together” (Practice Nurse 7, Round 2),* and *“So many of the patients are shared anyway, you know, they sort of bounce back and forth from the normal heart failure program, integrated care, when they have deteriorations, so we work as a team” (Hospital Nurse 4, Round 1).*

Good communications and upskilling of community healthcare providers were noted as key facilitators: *“We want to empower the GP … we want them [patients] to look after themselves of course, and to work on themselves, but through the GP” (Hospital Specialist 10, Round 1).*

The two hospitals implemented aspects of the program differently and interviewees sometimes confused pre-existing or related programs with newly introduced WSICP strategies:

*At Blacktown it’s set up differently; at Westmead we almost use it as a post-discharge clinic-we’ll see patients that aren’t necessarily suited in the program, but at Blacktown they like to recruit GPs first and then see patients through the GP (Allied Health 2, Round 1).*

#### Challenges

Interviewees identified numerous challenges to implementing integrated care approaches through WSICP. Subthemes described issues around roles and responsibilities of those working on WSICP; inter-organisational challenges; and challenges related to the scale of WSICP and achieving major changes with limited time and funding.

Early challenges included uncertainty about roles especially with new positions like care facilitators, but also difficulties for nursing, allied health and hospital specialist staff in working together on the program.

*I think there’s still uneasiness between the teams, in terms of integrated care working, I think we need to do a lot more teambuilding exercises there. I think it’s still very much viewed as an us and them approach (Care Facilitator 2, Round 2)*

Broader challenges described by interviewees often related to differences between the hospital culture and that of general practice: *“I wonder about…, this disinclination on the LHD [local health district] staff to recognise that community health or other services are of any relevance to this whole exercise” (Manager 3, Round 2)*, and *“…our culture needs to change a bit. I think general practice has been a bit of a silo” (GP 7, Round 1).* Interviewees frequently spoke about siloed provision of care which inhibited the sharing of information, even between hospitals. However, this was starting to slowly change as WSICP became more established: *“We were working in siloes before, almost didn’t know the other was there” (Hospital Nurse 4, Round 2).*

Inefficient IT was a constant source of frustration in its failure to bridge these siloes through better communication, shared records and efficient referral processes.

*Another frustration is the whole integration of health records; that’s been hopeless…the other frustration is they said we could get e-referrals. We haven’t got any e-referrals from any of the external practices…we think that GP’s should be able to e-refer; they still can’t e-refer to us at all (Hospital Specialist 1/Manager 7, Round 1)*

By the later interviews there was some evidence of health information being shared across sectors, although often as a result of WSPHN staff visiting general practices to build IT capacity, and care facilitators manually updating hospital records.

*They managed to link my Mum’s entire medical history with both Blacktown and Westmead, through the GP [via Care Facilitator]. So in emergency situations those places have full access at the touch of a button rather than me having to explain everything or try to remember all the details, or remember all her medications (Carer 17, Round 2)*

The WSICP was seen as influencing change through its support of Patient Centred Medical Home models of care, however, the fee-for-service remuneration of general practice was considered a barrier to team based care: *“Despite the small volume of incentives that we’ve managed to bring in with this program, the system is still geared to reward high throughput, but not high value” (Manager 3, Round 2).*

The size and complexity of the process of the WSICP transformation was a common theme: *“You’re rebuilding, changing, you’re realigning the way we’re doing business. We’re trying to turn the Titanic around a little bit and we’re slowly doing that” (Manager 6, Round 1).* Although the WSICP was showing promise, concerns were expressed that the limited time and funding for WSICP made it difficult to establish the program, change behaviours, and demonstrate improved health outcomes.

*We have to allow the time to get this message out to the GPs, allow time for changing behaviour…we’re not even a year into this and I think we’re trying to change a system that’s been in place for a very, very long time (Hospital Specialist 3, Round 1).*

Interviewees emphasised the need for long term commitment noting that retaining staff and maintaining gains already achieved would be difficult if the program ended too quickly: *“it’s going into mid next year [2017], that makes it very difficult people are worried about what’s going to happen after that, a lot of turnover of staff– then you have to start again, retrain people and fill jobs” (Hospital Specialist 3, Round 2)*.

#### Added value of integrated care

Interviewees identified many improvements to care as a result of WSICP. Subthemes described these as building capacity, education and upskilling of patients and health care providers; changes in practice; valuing WSICP; and suggestions for future practice.

From early in its implementation, healthcare providers, patients and carers valued the benefits provided by WSICP. Healthcare providers described services as more time efficient, and potentially more cost effective.

*Initially I thought it was going to create more problems, like take a lot more time. If anything it's actually made it work a lot more efficiently. And care plans are now up to date. Care plans are being followed up properly - I'm definitely seeing that it's helping (GP 9, Round 2)*

Patients reported support from multidisciplinary teams to self-manage their care and accessed care in the community that was described as holistic and patient centred, and included services outside the program. Interviewees said: *“I’m seeing a lot more improvements, in terms of the patient’s ability to self-manage their condition and making sure they go to their GP before it becomes worse” (Care Facilitator 3, Round 2)* and *“with the case conference I think it is a good thing, because we can see everyone’s input on it and we can work together as a team to better manage this patient” (Care Facilitator 4, Round 2).* When patients needed hospital care, patients and their GPs, knew who to contact and patients valued seeing people who knew them.

Multidisciplinary approaches upskilling and empowering patients (particularly in Rapid Access and Stabilisation clinics) were said to reduce the need for hospital admission: *“We’ve slightly decreased the readmission rate, so I suppose that’s something, as in they’re weighing themselves regularly, they’re watching their fluid restriction, they’re taking their medication. Maybe we’ve got some people to stop smoking” (Hospital Nurse 2, Round 1).* Another said: *“a patient who used to come in once every month, now haven’t seen him for a few months in the hospital because he’s been managed through integrated care” (Hospital Specialist 7, Round 1).* Patients learned how to manage their conditions more effectively and were making lifestyle changes: *“They’re brilliant, they explained what will happen, and how to deal with it (Patient 14, Round 2).*

Healthcare providers and patients appeared to collaborate more. The GP support line connected GPs with hospital specialists, while the patient hotline was regarded as providing a reliable contact point and improved patient access to the hospital: *“they get a number to call, they can just pick up the phone and ring the nurse or pick up the phone and ring the doctor… or go to the GP, get the GP to call us. I think that is a big plus” (Hospital Specialist 11, Round 1).* Patients and healthcare providers valued working with care facilitators who further helped connect them with others: *“I think they appreciate that there is a care facilitator as well - a bit of a one stop kind of shop if they have got questions or problems, help them navigate the system” (GP 6, Round 2).*

Healthcare providers discussed upskilling of the multi-disciplinary team. General practice staff described the education they received from care facilitators and also through case conferences, practice visits and evening workshops convened by hospital staff. They particularly valued case based learning approaches commenting *“they upskill GPs. I still feel anxious about starting people on insulin but I am able to do that now, whereas before I wouldn’t have felt comfortable doing that” (GP 5, Round 2).* Hospital staff also commented on learning about general practice: *“Going out to GP practices and doing some teaching has been incredibly eye-opening, I’ve got a much better understanding of what it is my GP colleagues want and need” (Hospital Specialist 2/Manager 9, Round 1).*

Collaboration and communication between hospitals and community based care providers were said to be improving: *“There are actually more GPs contacting the service with regards to referring their patients to our care. There are more doctors being talked to by the care facilitators in regards to how the services are being done” (Hospital Nurse 6, Round 2).* General Practitioners reported having better communication from hospitals: *“The doctors from the hospital are more into calling us for more information, there’s no hesitancy to ring us if they need help” (GP 8, Round 2).* Patients and carers described consistency in the care they received from different care providers. They valued the strong multidisciplinary team based approach of WSICP in both hospital and community, and perceived they had greater control and confidence:

*We are on the same track. The psychologists, the doctors, the care plan clarified – hopefully you get to understand drugs, how to manage correctly, so they are good. It’s also, like an assurance for me that I’m doing it right - as a carer (Carer 18, Round 2)*

The WSICP was described as keeping people well and treating patients earlier in their illnesses. A hospital specialist said: *“We’re picking up changes earlier and keeping on them, it does help them to self-manage a bit better” (Hospital Nurse 4, Round 2).* Rapid Access and Stabilisation clinics were central in this preventative approach and hospital staff sometimes provided home visits for patients: *“…since he’s come home there’s always someone ringing up or coming out” (Carer 19, Round 2).*

Most interviewees described positive outcomes from the holistic focus of WSICP. *“...they would see the diet educators and doctors all at once – so you can package the service into a one-hour, two-hour period, rather than, say, an admission or have a patient come back three times to see different parties” (Hospital Specialist 4, Round 1).*

Interviewees provided numerous suggestions for improving ongoing implementation of WSICP. Some spoke about extending access to those not currently meeting the inclusion criteria: *“it would be good to expand it a bit, it would be probably good to look – well, we’re doing chest pain and heart failure but to link in a hypertension clinic” (Hospital Nurse 2, Round 1).* This also included building nursing home capacity:

*Why can’t we go to the nursing homes and try and educate the nursing staff? If somebody is short of breath, rather than sending them into the hospital, they actually have qualified personnel there that can try and manage these things (Hospital Nurse 3, Round 1).*

Others suggested extending valued WSICP activities such as case conferencing in additional clinical areas and through use of videoconferencing*.* Greater access to allied healthcare providers was recommended, including provision of group sessions. *“A clinical psychologist - there’s a role for them. I’ve done my statistics and in my clinic about 50% of my patients suffer some form of mental illness, whether it’s mild or severe” (Hospital Specialist 7, Round 2).*

Promoting WSICP more strongly and continuing learning activities across sites and over time were other recommendations. *There’s not a lot of advertisement regarding the program, what we can do. I think you need a full project manager to help with communication, newsletters, you know, establishing who we are (Care Facilitator 2, Round 2).*

The biggest source of frustration in both interview rounds related to IT and communications, and interviewees frequently recommended a system enabling shared patient records by connecting hospital and general practice IT programs.

*The thing that would make a really big difference would be if we could look at their notes, and they could look, maybe not everything, but if I could actually look and see what's happened. If we had a shared electronic record (GP 7, Round 2)*

Interviewees further recommended co-locating integrated care services in the hospital, providing more space for this and improving access for patients to these services. Hospital staff said: *“the other frustration is that we don’t have our own team down there. I have an educator with me, I don’t have a dietitian, and I don’t have psychologists” (Hospital Specialist 5, Round 2).*

Further investment in general practice was also frequently recommended in comments such as: *“I think it needs a little more investment in general practice… we don’t have the manpower or staffing or the funding to employ someone to track these patients and recall them in” (GP 3, Round 1).* Additionally, interviewees identified the need to collaborate beyond the health sector for wider systems change: *“Urban design, transport, food supply and physical activity, and then identifying people at risk of, say, chronic disease and then working with primary care as they interface with the health system to keep them healthy and keep them well” (Hospital Specialist 8/Manager 7, Round 1).*

## Discussion

Our qualitative evaluation of the WSICP enabled us to explore the experiences and perspectives of a range of stakeholders at early and later stages of program implementation. We identified three key themes that described the set up and operationalising of the WSICP, the challenges encountered, and the added value of the program to health in western Sydney. The WSICP was a large undertaking that took time to establish. Delays were attributed to LHD “bureaucracy” and slow engagement of the primary health sector. Information sharing was impeded by IT challenges. Lack of clarity around staff roles caused concern to those engaged in the WSICP. However, over time these challenges were being addressed. We noted the commitment of staff in their efforts to implement the program. Impacts of the WSICP were described, including reduced hospital admissions due to improved patient self-management, greater communication and collaboration between healthcare providers across health sectors and an increased capacity to manage chronic illness in the primary care setting.

We focus our discussion on key findings of relevance to others seeking to integrate health services. These include consideration of the risks of undertaking such massive systems change as a limited term pilot project; the importance of patient centred approaches in joining up health care; and the need for integrated care to build on strong models of primary health care.

Transitioning health systems to more integrated models is a substantial task [35]. As with the WSICP, integrated care programs are sometimes set up as pilot programs with the aim of scaling improvements across the health system [36, 37]. System wide changes to processes and structures and targeted allocation of resources are required to achieve integration of care across various health sectors [38]. As observed in other healthcare integration programs [39], the size and complexity of the WSICP were noted by our respondents who, whilst agreeing that systemic changes were occurring, suggested that such change requires a sustained long term effort.

The WSICP implementation was delayed initially by bureaucratic processes, a challenge described in other settings [40]. Engagement of the primary health sector was slower than anticipated, and WSICP used care facilitators to assist in enrolling general practices. Attention was paid to upskilling of both hospital and primary healthcare staff, although orientation of new staff was initially not as well managed. As with other integration programs [41] our respondents described contributing additional effort beyond their usual work roles to establish the program. Interviewees were concerned about ongoing funding and security of employment after the program’s trial period. This posed problems in terms of attracting and retaining key staff.

However, sustainability and scaling of health system improvements can be informed by key learnings from pilot programs [42]. We noted that one of the Rapid Access and Stabilisation clinics had expanded services to include patients presenting with chest pain. Other suggestions for expansion of WSICP including engaging aged care facilities in the program, were recommended to further reduce unnecessary hospital admission.

Patient centred approaches are important in joining up health care and can reduce hospital admissions and costs [43, 44]. Facilitators of patient-centredness identified in other settings such as better communication across sectors [45, 46] and with patients [6], enhanced multidisciplinary collaboration [47, 48] and a focus on prevention and patient self-management [49, 50], were identified by our participants as key priorities of the WSICP. All participant groups valued the personalised focus of WSICP and particularly the access to holistic care early in the illness trajectory facilitated through the RASS clinics. Patients and carers had a sense of familiarity with hospital staff and could contact them at any time through a dedicated “hotline”, but were also encouraged to have their own GP as the main provider of their care. The care facilitators in WSICP often provided follow up of patients and linked them with other services outside the program. They also connected the GPs with the hospitals through their access to both hospital data and primary healthcare information including patient care plans stored in E-health records. This “connecting” role provided early and ongoing successes in WSICP until the IT systems built capacity for more effective sharing of information. Care facilitators were also active in promoting the value of WSICP and embedding new and trusting relationships, and this is crucial in fostering collaboration and breaking down insular healthcare provider attitudes [9, 51].

The need for managing chronic illness in the community is documented [49, 52], and we noted the importance of a well-supported primary care sector as part of an effective integrated care model. This included providing GP teams with information about patient management through case conferencing and consultation with hospital specialists in the RASS clinics. General practitioners particularly spoke about their increased confidence in changing medications and respondents recommended expanding case conferencing to other chronic illnesses. The WSPHN supported GPs to use communications and information technology systems.

Interviewees also discussed needs for improved support for general practice beyond the WSICP particularly the need for changes to the fee-for-service funding model of Australian general practice. Whilst this model of payment may suit care for acute short term conditions it is less suited to the ongoing care required for prevention and management of chronic illness [15, 53]. Some of our respondents pointed to successful integrated care programs in other countries and emphasised their focus on care quality rather than patient throughput. Current trials in Australia with the Patient Centred Medical Home and Health Care Home models of care are designed to better support quality primary health care with bundled funding including for management of an enrolled patient cohort [54–56]. These models of care align strongly with the WSICP and position the primary care sector as a multidisciplinary team leading integrated care which is delivered predominantly in the community [57, 58].

### Strengths and limitations

A strength of our research was the large number of interviews we conducted with stakeholders including patients and their carers, a wide range of health providers, and the management team responsible for implementing the WSICP. We gained a nuanced understanding of their experiences which was further enhanced by conducting two rounds of interviews over the course of the WSICP. With integrated care programs frequently failing at the implementation phase [25], this thematic analysis of integrated care experiences and perspectives, and our regular reporting of the findings to WSICP management, informed the ongoing roll out of the WSICP. Resulting program changes were explored in later interviews. This approach is likely to be of value in other integrated healthcare initiatives.

Although our approach enabled us to reflect on the overall experience of the program comparing earlier with later phases of implementation, we did not reflect on changes in the individual experience of the program over the course of its implementation. A further interview round at the completion of the WSICP, may have provided valuable insights into the sustainability of the program and additional recommendations for translation of the pilot into ongoing approaches. We were also conscious that each interview has a unique power dynamic that can influence the data that is collected. Differences in power can play out, sometimes implicitly, in spite of the interviewer striving to maintain a position of neutrality. The interviewer was responsive to this and we also ensured that our analysis was driven by themes that were agreed upon by the research team rather than a single researcher.

## Conclusions

The WSICP was experienced as an innovative and valued approach to integrating care for people living with chronic illness and as a large scale systemic change in the delivery of health services. Setting up and establishing WSICP was time consuming but ultimately the patient centred approaches of WSICP improved access to timely care, increased patient self-management and illness prevention, and reduced hospital admissions. The support provided to primary care enhanced the capacity of GPs to manage chronic illness in the community, in collaboration with hospital specialists.

Program sustainability will require a commitment to ongoing support that enables retention of key staff, effective sharing of information and continuing and expanding the key WSICP strategies. In the primary care sector, the current fee-for-service funding of chronic illness management will need to be reviewed to better support quality care from multidisciplinary teams led by GPs. Effective evaluation and timely feedback will be crucial to sustainability.

These learnings from the WSICP are particularly useful for policy makers and healthcare providers as healthcare systems around the world are increasingly challenged in providing care for chronic illness. Australia’s health system shares similarities with others and many of our findings from the WSICP are relevant in other healthcare contexts.

## Supplementary information

**Additional file 1.** Interview Schedules.

**Additional file 2.** Full thematic analysis.

## Data Availability

Raw data is not available for public access due to ethics requirements of privacy in place at the time of the initiation of this study. The authors declare that de-identified data supporting the findings of this study are available within the article and an additional file.

## Abbreviations

CCF: Congestive cardiac failure
COPD: Chronic obstructive pulmonary disease
COREQ: Consolidated criteria for reporting qualitative research
EHR: Linked Electronic Health Record
GPs: General practitioners
LHD: Local Health District
RASS: Rapid Access and Stabilisation Service
WSICP: Western Sydney Integrated Care Program
WSLHD: Western Sydney Local Health District
WSPHN: Western Sydney Primary Health Network
WSU: Western Sydney University
