# Supplementary material for: Key stakeholder experiences of an integrated healthcare pilot in Australia: a thematic analysis
Source: BMC Health Serv Res. 2020 Oct 7;20:925. doi: 10.1186/s12913-020-05794-2 (PMC7542969; doi:10.1186/s12913-020-05794-2)
Supplement: Supplementary file 2 — Additional file 2. Full thematic analysis. [file 12913_2020_5794_MOESM2_ESM.docx]

**Additional File 2 - Full thematic analysis**

**WSICP Thematic Analysis with illustrative quotations**

The following analysis of the interview data is structured within three main categories of: Set Up/Operationalising; Challenges; and Value Add. The key themes corresponding to these categories are provided in the table below. In the analysis, each of these key themes is further elaborated by subthemes which are then described and illustrated by selected quotes.

| Set /Up Operationalising | Challenges | Value Add |
| --- | --- | --- |
| Managing WSICP | Interorganisational Challenges | Building Capacity , Education and Upskilling |
| Promotion and initiation | Challenges with Roles and Responsibilities | Changes in Practice |
| Access to WSICP | Scale of the Undertaking | Valuing WSICP |
| Understanding Integrated Care |  | Suggestions |
| Unrelated Activities/Programs/Processes |  |  |

All participants’ voices have been presented in the analysis. Colour coding is provided to identify the stakeholder group and type of participant within the group. The following key provides relevant colour codes and the participant acronyms.

**Participant Key:**

MG (Management Group); HS (Hospital Specialist); HN (Hospital Nurse); AH (Allied health); GP (General Practitioner); PN (Practice Nurse); CF (Care Facilitator); PC (Patient/Carer).

*Black font indicates first round interview (noted as Round 1)

*Blue font indicates second round interview of first participant (noted as Round 2).

*Purple font indicates new second round participant (noted as Round 2)

| **Set up / Operationalising** | | | |
| --- | --- | --- | --- |
| ***Management of WSICP*** | | | |
| **Effort**   - *WSICP has created extra work in General practices and hospitals* - *Time taken in development of program including for meetings and afterhours* | * in terms of the online care plan, it’s created extra work for us (GP1 Round 1)  * Had lots of meetings and a lot of doctors still aren’t that keen because of the work involved but we’re getting there (GP5 Round 1)  *It’s more busy I guess but I’m still adjusting in working around things and I can still do my other jobs (PN1 Round 1)  *we have had a lot of input for it and not seen many patients actually enrol, so there has been a lot of time and money spent on introducing this for our practice…it's also been very difficult for us nurses, to be honest, putting it into place at a systems level because the admin it's so all over the place at the moment (PN3 Round 1)  *it was a long process getting those patients involved. It involved the whole practice, with reception staff, nursing staff, nurses and the GPs, and even also the care facilitator. (GP5 Round 2)  *The engagement enrolment strategy is tedious and consuming of efforts and time and – hard to know how much that has really helped the general practice. (MG4 Round 2)  *It is affecting the workflow because we have not got the time to sit down and physically go through all that information… we’re reviewing these plans normally anyway for the patient but we’ve got to do it in two places at once and time doesn’t allow that. (PN5 Round 2)  *It is hard for – let’s say we’ve got 12 patients booked in, because the consults I do I can see three or four patients, but the doctors might want me to see all the patients and it’s just not possible (AH2 Round 1)  * I’m working on it three days a week and there’s an enormous amount of work to do (MG5 Round 1).  * that’s been quite stressful because they don’t give you additional – it’s a set up approach. They have given us money to start, but before that, it’s a lot of hours put in of our own time and private time too to get this up and running (HS11 Round 1)  * I’ve attended so many meetings about this whole program over the time (HS1 Round 1) | | |
| **Bureaucratic delays**   - *Lengthy bureaucratic process as part of large organisations* | *I mean, we took about six/seven months to plan and then there were delays within the LHD because the LHD is a cumbersome beast and so positions need to be created, they needed to be created, they needed to be signed off and then we needed to go through a financial process. There's a huge amount of bureaucracy which is a massive bugbear coming from an NGO environment where it was a big organisation but nowhere near as bureaucratic (MG2 Round 1).  *hospital processes have held up the Integrated Care Program a lot, which I think has been frustrating, but that has slowed things down a lot even though integrated care was quite committed the hospital wasn’t yet, really together (HS1 Round 1)  * anything you put a job in for, if it’s anything that writing a [IT] program – I don't know how to write a program so maybe there’s quite a bit involved, but it just seems to take so long to get anything done (HN2 Round 1) | | |
| **Funding allocation**   - *Requests for more discretion in allocation of funding* - *Unequal funding distribution across sites* | * I don’t manage the financial end of anything, I don’t have much understanding of it but for departments to have more scope at grassroots level to decide where money is better spent, I think, is better than having people way up in higher levels of management deciding where money is spent - - - and a little bit more scope to say, okay, “Well, thank you very much for the funding but we don’t actually want it right here, we want it over here, and we don’t want it for two years, we want it for a bit longer because two years isn’t enough [laughs] to get it off the ground (AH6 Round 1).  * Respiratory were saying maybe we could come out and show you how to do spirometry. So and I think actually respiratory said that there’s not enough money to do that and it’s an expensive thing but anyway. So if more money can go into that, that would be good.(GP5 Round 1)  *we don’t have a dietician with us, it’s a really important part of the program, but funding is a limitation. One of the podiatrists is funded through Integrated Care – one and a half. (HS5 Round 2)  * Certainly the money that was given to Blacktown Hospital is half of what was given to Westmead Integrated Care (HS11 Round 1) | | |
| **Policies, procedures, management unclear/changing**   - *Hospital staff unsure of referral procedures* - *GPs unsure of referral* - *No set guidelines or criteria - leading to changes along the way* - *Guideline****s*** *still not clear by Round 2* - *Program outcomes unclear* - *Instability of senior staff engagement impacts program direction* | * I received an email saying, “When you see a patient on the ward that needs outpatient dietetic services and has diabetes, if they’ve got a green referral card they can go through integrated care, if they don’t have a green referral care card they go through the usual channels”. And I said to my colleagues, “I haven’t seen one of these green referral cards. Has anyone seen a green referral card?” and they’ve just gone, “I don’t know, I haven’t come across it” (AH6 Round 1).  *I was ringing up about an ischemic heart disease patient and Blacktown weren't set up to manage that there, they said Westmead's team is but Blacktown's wasn't, so they just advised to send the patient into the ED rather than use Rapid Access which is fair enough. But it seems like there was still some differences between what the two hospitals do (GP6 Round 2)  *things are changing – so criteria, information and guidelines for the actual clinic are still starting to come out, so everything’s not set in stone, everything’s not so much finalised yet. So there’s still areas that are changing, so I suppose you're adapting as you go along (HN2 Round 1)  *a discharge guideline has been put out in terms of discharging patients off the program, and that’s been a little contentious; I think practices are unaware or they may not be following the process correctly, but there’s been some dispute over the process and what needs to be done. So that needs clarification (CF2 Round 2)  *a very big issue – is that no one is clear on what the outcomes of the program should be, or what we should be showing to show it works. From the top down nobody thinks of a clear idea and what benefit we should be showing or where we should be aiming this at to show a benefit by February 2017 (HS11 Round 1)  *there’s been a bit of a lack in continuity with the senior management, and there have been a few people who have come and gone, so I’m engaging with some people who are extremely good, but now they’ve left, I don’t know where this actually leaves us (HS5 Round1) | | |
| **Evaluation and Quality improvement**   - *Active research and evaluation across the WSICP* - *Particular areas identified for evaluation e.g. no shows and discharge to GP* - *Evaluation considered central in providing care and needs to be embedded in systems improvement processes* - *Concerns re narrow focus on integration and lack of access to some data* - *Learning from evaluation leads to ongoing program development* | * Pretty good, actually, because you get to see what each speciality is doing and how many patients we’ve actually enrolled into the program, whether we will get more GPs and things like that, looking at data to see if we’re actually making a difference (HN3 Round 1).  *they've done it about three or four times now - where they go in and they do a bit of a quality audit to work out what's actually happening behind that referral and whether they're adhering to the program elements (MG6 Round 2)  *I’d like to say, yes, I think, it has been worth it, but I can’t answer scientifically until I have that data…and I’ve been working to accrue that (HS5 Round 1)  *I did institute a survey of GPs about what their educational needs are around COPD and then it went broader to diabetes and cardiology, and that’s been really revealing. And I need to start to implement what the GPs told me their educational needs were but I don’t know how to do it. (HS2/MG9 Round 1)  *identifying the reasons for no shows could be useful. We often feel it’s a shame when people don’t come here, because we offer such a good service – they’re missing out. (HS4 Round 1)  *one of the purposes is to try and see whether we’re able to – we haven’t evaluated yet, but I suspect that we’re discharging many more people back to the GP than probably what was happening in the past, so, that’s some of the stuff we’re trying to look at, so that we have some objective information as well (HS3 Round 2)  *We have got stats on it - they’re substantial, in cost savings and I can’t remember the amount of days, in-hospital days saved. (HN2 Round 1)  *There’s absolutely no evaluation of what is going on in the community, so what services are being used. Whether cross-sector supports are being brought into play, even allied health, it’s just a non-event. There’s an undue focus at the RASS end, and a complete neglect of community end; I think that needs to be fixed (MG3 Round 2)  *They’ve been collecting data for a while, we really questioned the type of data that they’re actually obtaining…around 30 face-to-face encounters with patients over the that month yet very few are captured because they’re just looking at the integrated care, they’re not looking at all of our productivity (HN5 Round 1)  *I don’t know. I mean, how do you audit the pathway? I mean, we hope the GPs use it, but there’s no way for us to know if they’re using it or whether the use of it has improved the health of patients (HS11 Round 1)  * It needs to be thought of as part of our regular care, not as research- - - it’s not research. I mean, it’s monitoring which is crucial, because, I mean, we do things like M&N meetings and they’re relatively all systematic and we need systematic ways (HS1 Round 1).  * Oh, evaluating this program, taking the good things from it, understanding where it’s not delivering us any benefits and modifying it over time I think is the only way to go. You can’t go back to square one and start again, we’ve got people engaged, we’ve got processes running, we’ve got to take the bits that work. That’s what pilots are all about (MG4 Round 1).  *We're getting - actually we are documenting and evaluating as we go along and publishing.(HS8/MG8 Round 1) | | |
| ***Promotion/ Initiation*** |  | | |
| **Initiation and set up**   - *Partnership and linking different organisational cultures was foundational to WSICP* - *Program implemented before design/resources ready to go* - *WSICP not effectively communicated to GPs* - *PHN supports GP implementation* - *Patient unsure of program status* - *Referrals increasing* | *Initially we were a partnership, so - trying to bring two cultures together WentWest and the LHD. So there was some work to be done to bring – so that partnership had existed for a long time. The demonstrator was the first real opportunity to put that to test in terms of “let’s get something off the ground as a joint – as a partnership and let’s work on it and let’s build it, let’s implement it”. So we brought two teams together from different cultures and that team had to become quite cohesive and work well together actually. That was a very early kind of challenge that required some attention (MG2 Round 1).  * A lot of redesign work that is happening at the moment I think should have been done way before the Integrated Care Program was actually implemented or done because I don’t think GPs like change all the time (CF3 Round 1).  *I would have thought that the step of communicating with the practices, to have been worked out from the beginning. It seems like it’s not and that surprised me a bit because that’s the whole point of this, to share the information (PN7 Round 2)  *they just told me, “Go and do this integrated care program.” And I mean [CF] and [CF] come out and talk to me, but at the moment because it's still not up and running because we can't give out care plans or anything like that we're not really doing anything (PN2 Round 1)  *real understanding, and it's very variable from one GP to the next, I don't think the GPs fully understand the integrated care program or what it is or what they're actually getting their patients to sign up for and I think they have different ideas. I think there is a real communication gap (HS8/MG7 Round 1)  *it’s lack of awareness in our area. Yeah, lack of awareness that they’ve got that service that they actually can use. Not to say that we haven’t had GP’s ring up, we have, but it’s few and far between (HN2 Round 2)  *[…] from WentWest has been good too, she’ll come out or answer phones, send emails, she’s very good with communication in that sense of coming out. The support’s been great from them (PN3 Round 1)  *because the program’s really started and they haven’t actually done anything with it. Well, funnily enough, I mentioned it to [CF], she just rang me an hour ago, because I've got an appointment at the cardiac clinic tomorrow morning – glad she rang because I forgot about it. Maybe that’s a sign it’s working [laughs] (PC4 Round 1)  *to start with what was the actual number of patients - we didn’t really have much of a referral basis to start with, but as I’ve gone along, there’s been more and more referrals coming through (HS5 Round 1)  *There are more GPs referring their patients to our care. A lot more patients are being referred to our service (HN6 Round 2) | | |
| **Marketing and providing information**   - *Challenges in promoting the program particularly to GPs and patients and also time taken in the hospital setting to promote* - *Hospital staff promoting WSICP to GPs and Patients including those who may not have enrolled GP* - *GPs rely on their staff and PHN for program knowledge and help* - *Uptake improving in response to promotion* - *Patients also learning of WSICP from GP and enrolling in program* - *Limited promotion to Emergency and other staff also unaware of program* | *That's going to get the message out about the value of the service by that sort of stuff, but that takes time (HN4 Round 1)  *I think if you ask anybody in the cardiology department, they would know about the initiative now, and we’ve got a huge department as you know (HS1 Round 1)  * The Breathlessness Clinic [laughs]. Yeah. I don’t think there’s that much available from my understanding. I’m only here for a short time but certainly – my three patients, certainly have said that primary rehab is really the – the main thing they’ve received in a very long time, and the GP, they don’t even know about it (AH5 Round 1).  * we had such a pool of heart failure patients and I’m trying to speak to everyone and talk to them about ICP. Now, that doesn’t always happen so, if I’ve got a long list of people to see I may not have that time to go through ICP with them so some of them are being missed and sometimes other staff are not as motivated to talk about ICP with them (HN5 Round 1)  * getting the word out there that it’s a new program, getting people to understand the program, understand the referral criteria, and actually receiving those referrals has been quite challenging (AH3 Round 1).  *I have personally done a lot of talks to the Blacktown area GPs. A lunch time session - talking to them, introducing the Integrated Care Program (HS7 Round 2)  *we always write a letter back to the actual GP, even if they’re not referred by the GP, just to say that we’ve seen your patient and we’re here, and at least we think that in their mind it helps them to actually be aware of us (HS5 Round 1)  * we do mention it to some patients. But we are a bit hesitant about that, because we don’t want to set up a scenario where they might go to another GP for that reason (HS5 Round 2)  *we’ve done a lot of things to improve enrolment. We write a one liner at the end of every letter saying that they’re suitable for integrated care and the GP support hotline and the details are on the HealthPathways, so, that’s one form of advertising, and then we flag the patient and that list is given to Wentwest so that they can look at which GPs are involved with those patients and target those Practices for recruitment.(HS3 Round 2)  *the nurse within my practice is certainly helpful, if I lack time, then I can pass on the communication barriers for me, I'm lucky that I have a nurse. There is also a WentWest facilitator that comes to help us and educate me, or inform me of what I should know about this program. (GP8 Round 2)  *It’s starting to pick up now so we’re starting to gain more patients, and I think that’s just through advertising the actual role and trying to get the information out there (HN2 Round 1)  *[HN] at the clinic told me about it. She said, "Interested in going on it?" I said, "Yes.” Plus, I thought it would be good – I’ve been there a couple of times when I was in and out of ambos and hospitals (PC4 Round 1)  *they [GP] put Mum onto the integrated care system. Prior to that we weren’t aware of such facilities available for patients, really (PC17 Round 2)  * it comes up on their record that this patient would be suitable for the integrated care program. I just have to give them a two minute talk, explain what the situation is. And everyone I've spoken to has agreed to now be part of it (GP9 Round 2)  *Certainly I promoted it quite heavily within the department in respiratory medicine, so I think we’re doing quite a bit and don’t really know if we will continue to do so in terms of advertising the program. But emergency is another area where I think more engagement from them would be good (HS11 Round 1)  *I still feel that doctors in the hospital aren't as well aware of the program requirements…that was probably one of the reasons where we did have difficulty getting in touch with one of the doctors at the clinic… I'm not too sure whether the other doctors in the hospital are as well educated about clinic access and things (GP4 Round 2) | | |
| **Orientation**   - *Workshops focus on referral* - *GPs need assistance to use PenCAT effectively* - *Limited information for staff at recruitment stage* - *Compared to initial CFs, new CFs receive full orientation and mentoring* | * I think for Westmead I'd have to say the collaboration is very good because we've started doing some workshops on how to refer patients to the integrated care pathway (CF2 round 1)  * we weren’t quite sure how to use PenCAT I think in fact it wasn’t even working properly on our system and on our data (GP5 Round 1)  *There was minimal information, so when I applied for the job, I was just trying to research what Integrated Care was. I found that quite difficult. There was minimal information on the website, as in the health website, New South Wales Health (HN2 Round 1)  * new care facilitators coming on board have a different route to orientation to the way I was brought in, quite more substantial orientation than I received 12 months ago (CF2 Round 2)  * I had a full orientation – had to go to different community services…followed that up with another care facilitator who actually mentored me through the process of how to do things – we had a schedule orientation for more than a month for the program (CF4 round 2) | | |
| **Time required**   - *Takes time to set up program and streamline practices* - *Takes time to recruit GP practices* - *Hospital staff note quality of work due to WSICP but also that it is time consuming* | *I think that there’s been a lot of people that have also individually felt that they’ve spent a lot of time (HS1 Round 1)  * because it was a new system, where we were having to do everything electronically and there was a lot of glitches in streamlining it and, you know, things like that we didn’t have the letters up and running yet. So that was a time – that was ending up costing us a lot more time and a barrier in terms of how many people we could see (HS3 round 1).  * It just takes time, I think. And, so, it takes time for the PHN to recruit enough GPs, such that the majority of patients that come to us can be sent back to an integrated GP. We don’t have enough of them out there to send back to (MG1 Round 1)  * we’re trying to do some really good work and some really important work and I’m really proud of the work we’re doing but it all takes time (HS2/MG9 Round 1) | | |
| ***Access to WSICP*** |  | | |
| **Eligibility**   - *Mental illness excludes* - *Cancer excludes* - *Only CCF included not chest pain* - *Dialysis excludes* - *Out of area excludes* - *Wider inclusionary criteria allows greater access* - *Patients discharged from program if inactive status* | * if there’s just a depression or anxiety or something as part of it then that’s fine that they can be included but I think if they’re developing – say if they’re in mild stages of dementia but they’re not that bad I think they’re excluded (GP5 Round 1)  * patient has diabetes but is diagnosed with cancer then they can’t really be involved in integrated programs (PN1 Round 2)  * I've got patients who have diabetes and a heart problem as well but the patient was not inclusion, because the patient had non-Hodgkin's lymphoma (GP13 Round 2)  * if the GP is seeing a patient with chest pain, more often than not, they would send to the emergency department (HS6 Round 2)  * patients with type 2 diabetes but they are on the dialysis - an exclusion criteria so they're not getting to the program but I thought these other patients who have got more complex medical problems, they should be probably better cared for during integrated care program (GP13 Round 2)  * Oh, one of the GPs who’s actually been involved with the project, has got a patient who lives outside the area and therefore can’t be registered. Is that acting bonkers or what? So I’m taking the straightjackets off (MG4 Round 1)  * It's quite helpful that there is a clinical metrics inclusion criterion as well. The majority of our patients who would benefit from the program are able to get in in that sense (GP4 Round 2)  * we had some issues with patients we had enrolled that were then unenrolled from the program, because they weren't active in the program, which I think is a shame because those patients still would have benefitted from being in the program even though they may not necessarily been very active (GP2 Round 2)  * If you send through a patient, and the patient is uncontactable after four or five times, there’s only so much that the care facilitator can do. That person needs to be off the program, it’s a waste of resources (CF2 Round 2) | | |
| **Delayed GP enrolment**   - *Concerns of not effectively engaging with GPs* - *Perception re selection of GPs for the program as a problem* - *Patients missing out due to slow GP enrolment* - *GPs too busy and forget to enrol patients, or cannot spend enough time* - *Most referrals from Inpatients* - *Enrolment not consistent across LHD* - *Enrolling GPs still taking time* - *Patients receive some IC services even if GP not enrolled* | *I don’t know if we’ve heard enough from the GPs to get them involved because certainly they’re not signing up to the program now. I feel like we might not have done our homework correctly is my concern (HS1 Round 1)  *it’s more from the GPs engaging in the service. We’ve got a hotline we use. That’s accessible from the GP’s service through a mobile number that’s on the website. I’ve only had less than five phone calls in the last six months through that hot line. So GPs are not engaging…the barrier is the uptake of the service by GPs at the moment (HS10 Round 1)  *a lot of my patients are potential candidates for ICP but the practices are not engaged (HN5 Round 2)  * we got 30 something patients in the program at the moment, but the doctor is not really focusing on Integrated Care and letting me know that these patients are eligible for it; a bit slack (PN2 Round 2)  *They’ve got a selective criteria training for GP so maybe if we open it up to all the GPs maybe they will get used to it (AH1 Round 1)  * There are people in the hospital setting who would be recruited but they don't have a regular GP or a GP that is involved in the program. So they're not being brought into the program, even though they're eligible. (MG3 Round 1)  *I’ve been really busy and the sad thing was, there were so many patients that were coming through that would have been eligible for this. But just because it wasn't there in my mind I didn't think to recruit at that time. (GP9 Round 2)  *I think they [GPs] just need more time with their patients really. Some of them are just rushing. (PN6 Round 2)  *it’s not having enough GPs at the start that have been enrolled in the actual program, so we were getting many of our referrals from inpatients (HS5 Round 1)  *the phone is just not ringing and there is just – in my opinion, not enough GPs enrolled in this area. There's been some work to try and enrol GPs and there's a lot more GPs enrolled in Blacktown (HS2 MG9 Round 1)  *It would be nice to think that we could get more GP input from a community standpoint, but I don’t know how long that’s going to take. I think it will happen but I just think it’s going to be really long and slow. (HN2 Round 2)  *In terms of enrolling patients it hasn’t improved. Even if we don’t enrol them in to integrated care, if they have a diagnosis of COPD we still see them (HN3 Round 2) | | |
| **Patient language and other difficulties**   - *Need for individual strategies to address barriers* - *Challenges where English a second language* - *Preference for health care providers who speak their primary language* - *Too ill to attend hospital* - *Clinic difficult to find* - *Dietary (and SES) challenges for patients* - *Patient difficulty with appointment and waiting times* - *Mail contact is inefficient* - *Patients too busy to attend* - *Financial concerns of patients including losing their job* - *Patients not using IT* - *Some patients need greater home support* | * There was one lady who was very claustrophobic and so she didn’t want to go in the lift so this was – she was from Breathlessness Clinic, which is another aspect of the program, but – yeah, so in the end we solved that problem by organising to see her down in pulmonary rehab that she could get to without going in the lift. (HS9 Round 1)  *there’s a bit of a delay with interpreters. Yes, we can get phone interpreters, but it is a lot better to have a physical interpreter in the consult room and you might wait longer depending on the language if it’s very obscure (HN2 Round 2)  * Some people would say that if anything, it’s an opposite barrier because then we – people are more likely to refer them to our sort of stabilisation service because they can speak other languages. They’ll go back to the regular specialist because specialists can’t speak that language. It’s harder for them to see their specialists in their community who can’t get an interpreter necessarily, so then they get sent to us (HS1 Round 1)  *it turned out because he’d spoken to us with an interpreter and he trusted us with the interpreter – he had no idea what we said and went back to his GP for more advice, and the advice was very contrary to the advice we had given him, so a difficult situation (HS5 Round 1)  * So I often do have conflict with patients who really want their family to interpret and we’re not allowed to go down that path (AH3 Round 1)  *the majority of our patients are Filipino, so they prefer Filipino allied worker because they understood them more and they can speak in their first language, if they go to a dietician or a diabetes educator that is not Filipino, they can – language barrier can be a problem (PN1 Round 1)  *Some of them I know feel that they’re too sick to come, some of them it’s too much effort to get back to the hospital, some of them forget, some of them misplace the timing and interpreters – ugh. (HS2 MG9 round 1)  * most of them, not everyone can find the clinic even for new services, everyone gets told to go to the university clinic and to come down here for a few things – and if they have a wheelchair then that’s another hurdle (AH1 Round 1)  *…food and eating healthy… I think a lot of it’s poor education as well, financial, like being from a lower SES background, not having the money to necessarily buy the things that – like fresh fruit and vegetables. (AH2 Round 1)  *we’ve changed to walk-in appointments to try and get them in with the 24-48 hour, even five day…A couple of times people have been discharged at 2.00 or 3.00 and been told to turn up the next morning and they haven’t…they’re probably still in bed (HN2 Round 1)  *I ask for a script he goes, no I want you to come up, you know, when he thinks it's time for me to come and get checked [COPD]. I feel he knows my anxiety about and that. His practice is so busy, I'm not sitting there for three hours, I can't. I get too worked up over it. (PC8 Round 2)  *I have problems with the constant mailing, now that; one, the cost has gone up; two, it's not an everyday service; and a lot of our patients we found were not attending because they weren’t getting their mail in sufficient time (HN5 Round 1)  *I had a business going at the time, that’s obviously gone now because of everything, but I just felt really crook at work, so I arranged to go and just drop in at lunchtime. Because it was my own business, I was a one-man band, so customer service morning and afternoon and everything.(PC4 Round 1)  *some people are really busy, they work Monday to Friday, and the GP is closed on the weekend so they can’t come and see GPs. (CF4 Round 2)  *Well, I'm on ten medications a day, or ten doses a day, something like that. It's a lot. I take three for emphysema and three for diabetes and one for the reflux and one for blood pressure, so that's ten. The costs of scripts are adding up a bit.(PC3 Round 2)  *in Western Sydney it's probably the cost of medication and seeing specialists and things like that. Mostly general practice is bulk billed. They're able to see their GPs but it's - in an ideal world if we had a hospital clinic for every type of speciality it would help greatly. (GP12 Round 2)  *I’ve had a couple under threat of being retrenched because of their leave that they’re having to take to go and see doctors and to come to our clinic appointments (HN5 Round 2)  *Mainly having patients that don't have any Internet or computers and things like that. There are still patients that aren't using technology very much so they're not going to be able to access the patient portal side of the care plan or anything like that (GP6 Round 1)  * …and he's alone… so organising meals on wheels is needed because he's not able to cook. And organising services like home care. (GP13 Round 2) | | |
| **Patients refusing/ not valuing WSICP**   - *Patients not prioritising health care and difficulty promoting to younger patients* - *Difficulty getting to the hospital* - *Difficulties communicating with the hospital* - *Patients happy with current level of care* - *Poor patient motivation* - *Patients fear loss of independence from too much involvement* | * I think one of the challenges is probably trying to explain to patients and get their – get them to, I guess, agree to being part of the integrated care program just in terms of the location that we’re in sometimes with a bit of noncompliance and lack of, I guess, health awareness. So some patients may not necessarily be that keen. The ones that I’ve had so far have been okay (CF1 Round 1)  *they feel like they’re well, so they don’t need that kind of support or prevention to further disease or complications, they’re not acting – yeah, they don’t care about their health (PN1 Round 2)  *patients who are older patients have time to talk to us, and they have time to listen, and they might be the patients who are going to the hospital as well - we can enrol, then they get more benefit, but I feel that younger people, it’s very difficult to enrol - promote them to service (GP11 Round 2)  * Patients don’t – aren’t communicated to about the necessity of attending them. They don’t see it as a priority and they find with Westmead hospital hopeless to get to anyway, but they don’t see the priority of it and they don’t see the communication, and I think the other thing is that they find it difficult to communicate back with us (HS1 round 1)  *More than half of them said, oh, this is not needed, what is this for, what you are doing is okay, we don’t want another person involved (GP10 round 2)  *she had all the education in the world, she saw every member of the team numerous times, which is probably actually more than what we normally would do for a patient. I feel like for her it was just her motivation; she wasn’t motivated to make the changes (AH2 Round 1)  *there's a few patients who are just, like, “I don't want to have to go and attend anything, I don't want to have to – I just, no, I don't want to be told what to do with my health care,” and they’re just really not interested, I don't know why, maybe they just want their GP to do it (PN3 Round 1)  *some patients just – they just don’t want to see so many people. Yeah. Normally those people are medically noncompliant. So I think it’s not the program problem, it’s just their personal perspective (GP3 Round 1)  *Some patients have really been anxious about getting into Integrated Care, they see it as another level of loss of control, and it’s really hard to actually convince them, no, this will be better for you, rather than, no, there’s someone else saying what they can't do (GP14 Round 2)  *I get told by my patients a lot the fact of having extra things to do or more people involved was actually a barrier. He wasn't interested in signing up because the last thing he wanted was more phone calls or more appointments (GP6 round 2)  *out of that diabetes thing [case conference], they actually had a couple of patients not come back to the practice because they didn’t like what they heard from those specialists.(PN2 Round 2) | | |
| **Transport and parking**   - *Missed appointments due to parking difficulties* - *Illness makes access difficult* - *Parking expensive but some discounts are available* - *Disability parking permit is a facilitator* - *Accessing transport is difficult but buses can be helpful when parking is difficult* | * Yeah, they miss their rehab sessions, they miss therapy appointments, they ring and say, look, I drove around for an hour and couldn’t get a car park, that’s why I didn’t come to my appointment today. So I think that’s quite disappointing (AH5 Round 1)  * The hospital parking is atrocious at Blacktown, yeah. I have to be dropped off at the door because there's no way in the world that I could walk from the carpark down to the hospital and I couldn’t walk back there either. I think, I honestly do believe that they need more closer parking for people, like, invalid people (PC7 Round 1)  *with the wheelchair, my sister drops me right out the front door and I don't move until she comes.(PC8 Round 2)  *a lot of them don’t have much family support. So they're somewhat isolated, then they have to book patient transport, which is often a bus. And some of them are worried, because they're on diuretics, that they don’t have enough toilet stops on the way to the appointment. A lot of them don’t drive, a lot of them can't walk very far [with COPD] (AH5 Round 2)  *It’s a nightmare at Westmead. You’ve got to pay for your bloody thing, and you've got to wait to get in there. It’s so big, that place. So many people going in there, and the distance from the main door to the ward, you need a bloody vehicle to get down there (PC2 Round 2)  *I’ve now got a critical mobility parking spot, so I can park fairly close now in a designated spot. (PC14 Round 2)  * Transport I find is quite a big one. If they can’t - I guess some patients can be linked to community transport but some don’t meet eligibility criteria, so they can’t be and they have to catch a taxi to go to their medical appointments if they don’t have any family or relatives, so transport can have an impact that they don’t turn up (CF3 round 1)  *we found out, that we could get a bus up to the hospital, and that saves me worrying about parking (PC22 Round 2) | | |
| ***Understanding Integrated Care*** |  | | |
| **Good understanding of integrated care**   - *Patient centredness that includes carer and family* - *Coordinated and continuous care* - *Systems to support including informational continuity* - *Multidisciplinary team based care* - *Bridging gap between primary care/ community and hospital care* - *Communication between health care providers important from patient perspective* - *Prevent hospitalisation* - *Linking all services including in hospital and into community and beyond health* - *Building GP capacity* - *Mainly for complicated patients/ “frequent flyers”* | *It’s improving the care continuum with the patient. It’s got different elements; it’s about patient-centered care; it’s about integrating all the care providers plus that patient into their own clinical care plan. It’s about systems as well, so the systems around to enable that interaction to occur; we need to work together and I think that’s being integrated (HS1 Round 1)  *In a nutshell I believe that’s what the integrated carer system is about - integrating three different groups of people, which is hospital, GP, the patient carer or a different family member (PC17 Round 2)  *Like a one stop shop (PC3 round 1)  *somebody communicating with all the various specialities that take care of a patient. So, there’s one person kind of overlooking it all and making sure everything is working well together (PN7 Round 2)  *it means coordinated care. That’s the way that I like to see it. And coordinated from the perspective of the medical services, allied health and taking into account what the patient’s goals and objectives are (GP1 Round 1)  *main benefit for the patient is that there's continuity. Their healthcare plans are uploaded so other health professionals who are involved in their care can have an idea of what's going on with what other people are doing (GP12 Round 2)  *To me integrated care is between your doctor, hospitals, your specialists and whoever else is involved in the medical (PC7 Round 1)  *Care that’s shared between people in looking after patients, so we all know what’s happening and if you think a patient needs extra care in an extra area – or extra support, it’s easy to get in and get referred to those people (GP14 Round 2)  *encompasses all the people involved in that patient's care, so multi-disciplinary team to care, as well as being coordinated, so making sure there is good communication involved, and also involve the patient; so the patient is actually involved in their care as well.(GP6 Round 1)  *it’s communication with the doctor, particularly, for doctor to referral - and they interconnect. They interconnect - it’s not complicated, it’s simplicity. (PC18 Round 2)  *But, integrated care needs to be more general, it should be an approach, not necessarily a specific model (MG2 Round 1)  * its a team based approach where people are working in those various settings understanding their role. So there's not a transfer of care. It's more a contribution of a shared care approach between a hospital setting, a community setting and then even in a broader sense working with councils and transport and other sectors to keep people healthy (HS8/MG7 Round 1)  *Shared-care, basically. So, we’re looking at a group of people to look after the one patient. (PN5 Round 2)  *I think this is a seamless process integrating care between the hospital, GP setting and the community. I think that's what it's about for me and having a multifaceted, multidisciplinary team approach to patient care. So that's what integrated care is for me. (CF2 Round 1)  *My understanding it’s where they – together they talk to each other to ensure that she’s getting the right care for all of her issues. Not just focussing on one element (PC1 Round 1)  *it’s integrated care between general practice and the tertiary hospital system and trying to integrate the care of the patients across that system with, I think, an end goal of trying to – and have skills in primary care and it has care delivered in primary care but the end, end goal of trying to prevent hospitalisations (HS2/MG9 Round 1)  *GP and the health facilitator that is involved in the program, specialist if needed, to allow more care and support services to be given to the patient to help keep them at home, if possible, and with care in the community, and avoid, or prevent them from being admitted to the hospital as much as possible. (GP8 Round 2)  *broadly, for me integrated care is actually looking at improving the coordination in the patient journey – and it's not just through the hospital but through their whole health (HN5 round 1)  *We want to empower the GP and we don’t want patients to – we want them to look after themselves of course, and to work on themselves. But through the GP (HS10 round 1)  *It’s mainly for the complicated patients, so we can get everyone involved and everyone will know the patient’s situation, if there is any change. Yeah and also we know about who is involved in the patient care so we will know who we should talk to if there is an issue (GP3 Round 1)  *they’re looking to target the group of higher flyers in emergency departments who are not being well managed and link them in so that they can improve the comprehensive care (GP7 Round 1) | | |
| **Limited understanding of WSICP**   - *Varying understanding noted* - *For some, focus is on preventing ED attendances or on sharing information* - *Some describe lack of understanding prior to WSICP* - *GPs perceived to lack understanding of the program* - *Patients describe getting additional assistance* - *Patients may be unaware they are receiving integrated care* | *Understanding of some of the concepts are quite varied and very difficult to sometimes get people to engage (HS1 Round 1)  *Integrated care meaning that we’re actually looking after the patients in the community so that we’re, sort of, like, preventing over-population in [laughs] emergency, I guess, overcrowding (PN1 Round 1)  *Well, as far as I can see, it’s that they share all your information. That’s all. (PC12 round 1)  * this has been a real thing in the practice because no one seems to understand what it was. My understanding is now that if we have patients with those chronic illnesses like the diabetes and hypo and stuff like that, if they're not well and they need rapid access we can contact the specialist at the hospital (PN2 round 1)  * to be honest I didn’t really know much about integrated care before the program was introduced to this practice, didn’t really know anything about it, so I’ve only learnt since our surgery’s been put into the pilot (PN3 round 1)  * I didn’t know what integrated care was until it was explained to me. (HS10 Round 1)  *They get people from outside to try and help you – people that know what they’re doing to help you out, try and help your condition and try and help you work out and get over what you’ve got. Things like that (PC10 Round 1)  * I don’t know how much the patients know that they’re actually in Integrated Care. They just think it’s business as usual, that’s how it always ought to happen (HS8/MG8 Round 2) | | |
| **Differences in models across WSICP sites**   - *Discipline specific differences in templates seen to be inefficient by some and positive adaptation by others* - *Differences in use across hospitals and in services offered* | * I think within our hospital systems, like all the clinics work really differently. Like heart failure clinic is so different to diabetes clinic or COPD clinic. The letters that go out to GPs, the templates are also different. Like, some would have more information, some would have less and again, that’s a barrier because it doesn’t reflect that our side of the work is really efficient or consistent. It’s such a mixed approach and Westmead is so different to Blacktown as well (CF3 Round 1)  *Because they’ve all get their set minds and set agendas on how the model works (CF1 Round 1)  *there's a number of planks that we’re trying to institute in Respiratory as our version of integrated care which is a little bit different to some of the things that the other subspecialty disciplines are doing. (HS2 MG9 Round 1)  *at Blacktown it’s set up quite differently; for example, at Westmead we almost use it as a post-discharge clinic so we’ll see patients that aren’t necessarily suited in the program, but whereas at Blacktown they like to recruit the GPs first and then we see patients through the GP (AH2 Round 1)  *I was ringing up about an ischemic heart disease patient and Blacktown they weren't set up to manage that there, they said Westmead's team is but Blacktown's wasn't, so they advised to send the patient into the ED rather than use the Rapid access which is fair enough - seems like there was still some differences between what the two hospitals were doing (GP6 Round 2) | | |
| ***Unrelated Programs/ activities*** |  | | |
| **Specific non-WISCP programs**   - *Some programs and initiatives have predated WSICP and have been funded externally from WSICP* - *Integrated care has merged with WSICP* | *We've had one service, which you've probably heard about, with an endocrinologist coming out and working directly with GPs and chronic patients and building capability in not just the general practitioner but the practice team. That is a model that we would like to build here as well on other fronts, but you've got to have a specialist who is able to do it and has an understanding of the need and how to approach it (MG3 Round 1)  *the Spirometry Roadshow I think is helpful. We’ve only been to a few general practices and really that hasn’t come from integrated care, that’s come from a variety of drug reps saying to us, “Can you come out to this GP practice and give us a talk? (HS2/MG9 Round 1)  *the breathlessness clinic is a hybridised model from the UK. All of the rest of it is a lot of what Western Sydney wanted me to do. (HS2/MG9 Round 1)  *I’ve been involved with the rehab unit, on the program and also on the maintenance side of it since 2014, been going to Blacktown Hospital (PC9 Round 1)  *The integration of the chronic care management program and the demonstrator has occurred, which we love them both. So those programs are now the one program; the demonstrator being a bit of a subset of it…and we've retained the enablers parts, particular to the demonstrator (MG6 Round 2) | | |
| **Relationship with WSICP**   - *numerous programs parallel WSICP and share patients and staff* - *Challenges of harmonising WSICP and non-WSICP strategies* - *Utilising external providers that are not part of WSICP* - *Engaging with other non-WSICP programs to provide for complex needs* - *Programs designed to prevent illness expand upon WSICP* | * So many of the patients are shared anyway, you know, they sort of bounce back and forth from the normal heart failure program, integrated care, when they have deteriorations and so for, so, yeah, we work as a team (HN4 Round 1)  *we’ve set up an Acute Exacerbation COPD Clinic, more a stabilisation than rapid access clinic, where we see people following their hospitalisation, about two weeks after, to try and get them into pulmonary rehab or try and make sure that they’re getting better, and make sure that what was planned on their discharge summary has actually happened (HS2/MG9 Round 2)  *I also find the people that we don’t necessarily have enrolled in Integrated Care or aren't yet identified as needing integrated care, that if we have them in our pulmonary rehab service and they get sick, we’re then able to refer them to the rapid access clinic, and get them seen that way and that’s been really efficient (AH3 Round 1)  *I’m not sure if it’s from integrated care or not, but home visits I think are done by [CNC] who is not part of integrated care, but I think that perhaps that service, I don’t think its mainstream (HS10 Round 2)  *That was the heart failure clinic, but this is the cardiac rehab unit where you do physical exercises and treadmills, coffee grinder, under supervision and the maintenance programs are voluntary (PC9 Round 2)  *we're running two parallel initiatives; one is the Western Sydney Diabetes Initiative and the other one is the demonstrator program and it's of no surprise that there are elements that are in both those programs that overlap and reinforce each other. But actually harmonising and maximising the way they work together has been a bit of a challenge (HS8 MG7 Round 1)  *there’s also the case conferencing as part of the – what’s going on at Blacktown as kind of an almost separately funded process, which is focusing on case conferencing rather than the integrated care project, whereas at Westmead, we’re trying to do it as part of the integrated care project. I think that’s where some of the difficulties are coming (HS3 Round 2)  *we’ve got linked in with a private heart scan at Merrylands which has got a really good scanner for coronary CT angiograms. (HN2 Round 1)  *I don't know if this is part of the Integrated Care Program for a pharmacist to come to my home and see how my medications were being stored and double checking how I was using them and that sort of thing. (PC3 Round 2)  *We know in our patch, like Mount Druitt, they live in food deserts, so the food environment is not good and the affordability of fresh vegetables is not, and then they don't know what to do with them if they got them. So that's why we're working on urban design, food supply, identifying people at risk and getting them into lifestyle coaching programs like the Get Healthy phone line and there's a diabetes Get Healthy phone line (HS8/MG7 Round 1)  *The foot clinic, we have. And that’s another good thing too. Foot clinic’s started, fully fledged foot clinic. (AH1 Round 2) | | |
| **Challenges** | | | |
| ***Inter-organisational challenges*** | | | |
| **Inconsistent processes, procedures and policy across PHN/ LHD**   - *GPs need to adapt to different processes cross LHD* - *Challenges of meeting GP needs in context of hospital workload* | | * trying to link in with the three, sort of, sites, so the WentWest, the Blacktown and ourselves, trying to link in a little bit better so we can all see what’s going - because I don’t think there’s a wrong or a right way to do this, but it would be good if we were all, kind of, doing it the same way, especially for the GPs as well, they’re out there going, oh well, Blacktown does it this way, so I’ve got to do this, Westmead will do it this way, so I’ve got to do it this way (HN1 Round 1)  *the registrar during the day might be on the phone to emergency sorting the patients, so there has to be some understanding as to the workload that the tertiary institution takes on, we have to be mindful, too, if the GP has got a patient in their practice and they need to find out something about some information or anything, we need to have a reasonable time frame where that phone needs to be answered, so I think there’s some problems and some tweaking with that (HN2 Round 2) | |
| **IT systems**   - *Electronic communications is not working* - *Poor Connectability of hospital with community* - *Poor integration of health records* *but some carers note sharing of their medical information* - *Unsure if communication by providers has been received* - *Relying on mailed paper communications* - *Changes made in Linked-EHR are not flagged* - *Poor IT affects data collection and evaluation* - *Problems with numerous individual IT programs within the hospital system and General practices* - *IT issues impacting on data collection and program evaluation* - *Limited spending on IT* - *Using IT can be time consuming* - *IT improving but not all GPs have IT capacity* - *GPs not aware of Linked-EHR* - *PHN assists GPs with IT* | | *the software, it’s clunky for everyone. It's a third party system for us. It's a third party system for the hospital. It's not conducive for daily use. I'm not sure what could be done about that, unless we're all on the same platform (GP2 Round 2)  *there's been an enormous amount of work that’s gone on to link the GP IT system to the hospital IT system and I understand that that’s difficult, and it still hasn’t happened (HS2/MG9 Round 1)  *the care plan is uploaded to Top Bar and that. At the moment our Top Bar not working. So still working on all of that, so even care plans haven't been uploaded at this stage.(PN2 Round 1)  *it's still very basic at this stage. I think it's still the connectivity, I'm not really happy with. Ideally everything should be live and whenever we make a change, it should change instantaneously. (GP12 Round 2)  *Another frustration is the whole integration of health records; that’s been hopeless. I suppose, the other frustration is that they said we could get e-referrals. We haven’t got any e-referrals from any of the external practices. I mean, we think that GP’s should be able to e-refer; they still can’t e-refer to us at all (HS1 Round 1)  *… they managed to link my Mum’s entire medical history with both Blacktown and Westmead, through the GP. So in emergency situations the facilities of those places have full access at the touch of a button rather than me having to explain everything or try to remember all the details, or remember all her medications. (PC17 Round 2)  *encrypted electronic communication’s a big barrier, because a lot of people don’t use encrypted email, and we would love to just have everyone on an encrypted email and not have to be scanning thousands of paper documents in every week… (GP7 Round 1)  *for me to know that what I’ve sent has been read and acknowledged, and that’s important because I’m just sending it out into the ether, not knowing what the heck is going on and just hoping that the person on the other side has actually read it and actually acknowledged it (HS5 Round 1)  * the letter back to the doctors is still by paper mail, and we can’t ensure they’re on the common system. (HS5 Round 2)  * she [CF] started making changes on Linked-EHR which I can see, but I just don’t get notification of it – she told me she’d made a change in a page of Linked-EHR file, and that that was the only reason I knew about. She said to look out for it, so once I went in I saw it (PN3 Round 2)  *one of the major things is eHealth and the fact that we don’t have it, and different GP practices have got different computer programs, they don’t marry up with the computer programs that we’ve got (HN2 Round 1)  *some of the allied health don't have the same computer programs if we’re having to fax ….(PN3 Round 1)  * I think they need to be aware that the data collection is flawed and that’s how the data that’s collected, especially PIMS is how the program is going to be judged. So ensuring that works well and data collection is critical and I think it needs to be urgently reviewed (HS11 Round 1)  * trying to put everything electronically was to be able to easily pull data but we’re not able to do that. So, we’re trying to just keep our own audit, trying to look at comparisons – at the start of the clinic versus the end of the clinic for patient outcome, and looking at referral sources and also looking at discharge destinations (HS3 Round 2)  *there was a huge investment going in, that’s only huge in terms of limited PHN resources. In terms of the kind of investment that goes into developing IT systems more broadly, it’s very, very tiny, and the Linked-EHR suffers from lack of investment generally (MG3 Round 2)  *our nurses have been uploading them [care plans] onto Linked-EHR and it takes ages. It takes quite a long time for that process (GP5 round 2)  *we do drag a lot of information out of PenCAT. But because everything is not put on the computer it’s double the entry, and this is where we’re finding the major issues-we’re not fully computerised, is another matter. (PN5 Round 2)  *I would say 70% better. It’s working a lot more efficiently with care plans, I think, 90% of my patients have a Linked-EHR care plan which is great but for some practices it’s still an issue –whose IT systems are so fragile that they can’t cope with another software with Linked-EHR (CF3 Round 2)  *the doctors here, if you said to them you can find so and so’s care plan, or whatever in Linked-EHR, the first thing they’ll say, “Is what’s Linked-EHR”? (PN2 Round 2)  *we haven’t had many problems. I usually call the technicians from WentWest if there’s any problem in the Linked-EHR if we’re going to upload, it has been okay lately (PN6 Round 2) | |
| **Siloing and non- collaboration**   - *Hospitals don’t integrate well with community health care* - *Difficulties referring between hospitals* - *Interdisciplinary collaboration lacking* - *Different paths to common goals* - *Culture change needed in General practices* - *Poor relationships between GPs and Hospitals* - *Historical difficulties between hospital and GPs* - *A feeling that GPs may not consult through fear of being seen as unskilled* - *GPs not respecting CFs* - *GPs lack time for hospital specialists* | | * What goes on here in the hospital is fairly insular and we don’t necessarily – I’ve explored, through integrated care, community dietetics, what's out there in the community, which I’ve never really had the chance or the inclination or the push and shove to do in my inpatient role. (AH6 Round 1)  * I wonder about the optimal distribution of roles between the various care coordination functions and, in particular, this disinclination on the LHD staff to recognise community health or other services, are of any relevance to this whole exercise (MG3 Round 2)  * Westmead Hospital IC has a blood pressure monitoring system and I had a guy that I felt would benefit from that and I discovered that I can’t electronically refer to Westmead. In that sense the two hospitals don't speak (HN5 Round 2)  *I suppose also about us all sort of being more on one page, as opposed to I fix the foot and you fix the knee and someone else fix the heart… I suppose there’s more siloed care that we’ve been increasingly delivering over the years (HS1 Round 1)  *I think there’s still uneasiness between the teams, in terms of integrated care working, I think we need to do a lot more teambuilding exercises there. I think it’s still very much viewed as an us and them approach. (CF2 Round 2)  * I mean, in terms of chronic disease I still don’t think we’ve truly embraced the multidisciplinary approach. I still think that we’ve got the departments together but they’re still very much three different departments (MG2 Round 1)  *I think our culture needs to change a bit. I think general practice has been a bit of a silo, and I really like working with allied health people, it has a lot of podiatrists and physios and we communicate back and forth, so I really enjoy the team approach to patient care. (GP7 Round 1)  * its them [GPs] that’s not coming back to us. I think it’s really time for GPs to get their act together. If they want to benefit in this then they need to start making use of it and being more proactive in getting in touch with us (HN4 Round 2)  *GP practices being businesses sometimes it’s just quicker for them to see 15 patients in 5 minutes or 15 minutes, rather than put in all this work to have case conferencing and working together (CF3 round 2)  * Westmead Hospital and GP’s haven’t always had a good relationship, so there’s been a breakdown or a barrier there with starting the service from the word go (HN2 Round 2)  *I don’t know whether GP’s think if they ring and ask for advice or support we wonder what they’re doing? Did they get their medical degree where? I don’t know, if they think that and that’s why they’re not using the service, which is silly because, yes, they’re GP’s but they’re not going to be experts in cardiology, or respiratory, or endocrine (HN2 Round 2)  *I went up to a GP late last year, sent him all the information, he went to the appointment, kind of fobbed me off. He literally spoke over me and said send me information, no consent. (CF2 Round 2)  *I recently made a call to a GP - a GP told the reception that he didn't have time to talk to me. I have sometimes left messages for GPs to call me, but most of the time they don't call me back. (HS7 Round 2) | |
| ***Challenges with roles and responsibilities*** | | | |
| **Unclear roles**   - *No role definition or clarity of role expectations for CFs* - *CF role developing and clarity improving* - *Lack of role clarity influences care hospital staff can provide* - *GP staff unsure of their role in the program* - *Staff adapting their roles* | | | *But, again, it is a fairly new role as well and we’re pretty much the guinea pigs at the moment. Trialling. So, yeah. I guess at the moment we don’t really have clear definitions of what is expected or what is part of this role and what’s not. (CF3 Round 1)  *Well, that’s the problem. Because I think we were just sent out as crash test dummies to try and figure out what was the best way to engage GPs and patients into the program. You’d have to find a way to get an in to where they saw your role within the program. How did you complement their practice? Did they want you there at all? Were we taking up their time that they could be seeing patients?(CF1 Round 1)  *the boundaries between care facilitators and our team has been a bit shady in the beginning and that’s been a cause of problems because there could be boundaries that are unclear and there can be conflict and confusion. Certainly our nurses not being allowed to call patients after they’ve been seen in clinic and I think that’s a problem because the patient knows the nurse but then they’re going back to the GP and home. We understand that it needs a process, so I think that’s been a problem that’s been highlighted to me. (HS11 Round 1)  *it’s a bit more defined what we’re doing now. I think it’s a combination of consulting and actually co-coordinating or case managing (CF3 Round 2)  * it’s not entirely clear what I’m actually supposed to be doing, particularly with the inpatients because, you know, most of them are under the care of a respiratory physician so I don’t really want to come along and tell them, “You should change their puffers.” (HS9 Round 1)  *you’re employing me in this job but you’ve really hindered me because I can’t actually do anything here (HN5 Round 2)  *as a dietician I’m not sure if I’m supposed to get in contact with the facilitators. I think, again, that’s what it all comes down to; it’s such a new program no one’s quite sure of the role.(AH2 Round 1)  *I'm a bit confused because I'm not really quite sure what my role is in the integrated care program. (PN2 Round 1)  *There has been no defined role. I basically see my role as bringing the funding across to support other patients and support the program at [named] Hospital, and I’ve obviously delegated [HS] to run it with the help of the CNC and the clinical medical officer, but providing close advice and support for [HS], but a clear role for me in the program has not been defined and I think every department has been managing it in its own way(HS11 Round 1) |
| **Difference in employer/ responsibilities**   - *Responsibilities not matching role expectation of CFs* - *Hospital staff regard work load expectations as unrealistic* - *Admin not seen as clinical responsibility* | | | * when the principal GP has said I don't want to participate. It's up to and the clinical lead here to go out, have a meeting, discuss with them what's the problem and then cut ties or offer the GPs some support. (CF2 Round 1)  * the care facilitators were all about being in the practice with the GP, helping the GP identify the patients, doing risk stratification of their patient population and then helping the GP with their care planning and all that. I’m calling that the back-end. They’re also meant to be doing the front end which is working with the GP and the patient to help coordinate the patient journey (MG6 Round 1)  *for certain practices it depends on the GP and it depends on the practice and how much they’re willing to use us (CF3 Round 2)  * I know that the expectation is that we see 16 patients in our clinic session, I’m sorry, but I’m never going to see 16 patients within that timeframe…there's this disparity, what is ideal and what’s achievable (HN5 Round 1)  *the admin people don’t understand the real life of a clinician, and keep wanting me and my staff to do extra admin crap, and I will not do it - that’s driving me nuts (HS2/MG9 Round 2) |
| **Carer burden**   - *Caring is a fulltime job* - *Confusion* - *Need for information from program to help carers manage better* - *Use of WSICP can assist with costs of care* - *Grief and loss* - *Watching loved one deteriorate* - *Giving up work* - *Support from other carers* | | | *I am the one who looking after him, for the medicine, for the food, for the – everything. Even I'm working, I have to prepare that in the morning. And then I have to get - give that to the carer. Every time, an hour, every hour I call in the house…24 hour must be having somebody looking on him. (PC21 Round 2)  *Full-time. You know previously it was just a lot of hard work, I mean, and come home [from work] and you’d get all the stuff prepared before you go but now you know she’s 89 and I need to be there fulltime. (PC17 Round 2)  * I thought the actual insulin was to be – doled out - like it would help. I thought well this pill they need to give us 25 in the morning and 40 at night. It’s not like that. With the readings you’ve got to adjust it. And there’s nothing that tells you how much to adjust (PC22 Round 2)  *Well its rather hard being his carer, just after any information when he’s sick or that, it’s just hard to find information. I’m just totally confused, don’t ask anybody anything because they won’t tell you (PC19 Round 2)  *We got discharged, we got nothing. Lucky enough he has gone through it all before and I knew that I had to restrict his fluid intake, and watch his weight and all that, because they didn’t have much to give you when you left (PC19 Round 2)  *there’s been no real interactions since [husband] had the defibrillator, because that was major, really, really major, and really, really overwhelming, to be honest. We’d certainly heard of people with pacemakers and so on, and even now it is still something that remains a bit of a mystery to us, scares the life out of us. But, I don’t think there’s been much follow up from the program that way (PC13 Round 2)  *this worries me with some specialists, not all of them charge through Medicare. Privately some of them charge of the expenses, we are only pensioners (PC18 Round 2)  *another area of cost, his medication. You know, some months – depending how many Webster Packs, so it depends how often they draw your prescriptions down and how many, one month he could pay $50.00, and another month it might come down a bit or it might go up more (PC19 Round 2)  *the problem has been mostly for both of us, psychological, one for [husband] really understanding still how, where and why, and mine, yeah, grieving and changing lifestyle, I suppose, in a man who is still trying to be the man that he was, but can't be. (PC13 Round 2)  * The problem is, he's doing, not the same like before. It’s like I have a baby now [after stroke]. (PC21 Round 2)  *in 2013 I gave up full-time work purely because of me being single and with the type of work I was doing, I was travelling a lot interstate. And my concern was she was quite okay being alone at home, but you do sit back and think what if they call us or what if family does (PC17 Round 2)  *so suddenly you’ve lost your social support group in a sense, and now we’ve even lost our lifestyle to a degree.(PC13 Round 2)  *look, I spend a lot of time at home and that frustrates me, because when you’re confined to just your home all the time it’s like what’s my life come to? (PC17 Round 2)  *it’s good to everyday talking to carers because we also are with this problem. We try to look after our patient but at the same time sometimes we overlook ourselves (PC18 Round 2)  *I don’t know what support is out there for a carer because I’ve never looked, I’ve just put my head down and done what I’ve had to do (PC16 Round 2) |
| **Carer Supports**   - *Family support* - *Helping carers understand what to do* - *Suggestions* | | | *my son helping me. I feel I have somebody can help me when I go out to work. (PC21 Round 2)  *No other family care for her except me (PC15 Round 2)  *our GP asked us if a professor could come from the hospital to have a look at Bob. At the practice. And truly he just completely upset the program. I was very angry actually, because I thought well we’re doing good. I’ve got the opinion that if I can keep [husband’s] readings under 10 I think we’re having quite a good day. (PC22 Round 2)  *You’d go cross-eyed looking at all the bloomin’ wee-tiny writing on goods, to see how much sugar is in things. I’m 70 next month and don’t see too well (PC22 Round 2)  *the only problem I have at the moment is the dental. I know the Medicare has cut off everything so that is now our problem (PC18 Round 2)  *perhaps with the patient’s consent, a family counselling information session may be made available if they are receptive (PC13 Round 2)  * Communication, maybe groups, I don’t know whether there’s carers groups out there or, I don’t know. Just someone to talk to sometimes. (PC19 Round 2)  *I would definitely like to get involved with is if there’s any carer forums so the professionals – they won’t fix your problem, but that’s not what I’m expecting, but they will probably tell things that I’m not aware of, how to deal with different situations, whether the forums have group sessions where you can also talk to other people who are in similar situations. (PC17 Round 2) |
| ***Scale of the undertaking*** | | |  |
| **Size and complexity of program**   - *A change at systems level* - *Unexpected complexity* - *A large administrative burden enrolling into program* | | | *You’re rebuilding, changing, you’re realigning the way we’re doing business. We’re trying to turn the Titanic around a little bit and we’re slowly doing that. (MG6 Round 1)  *At the start of this, we really thought that integrating the health – the e-health systems would happen. But one of the biggest frustrations is that that has not happened, and I suppose though, maybe it’s because it was a more complex beast than we potentially contemplated. (HS1 Round 1)  *my mind boggles at the bureaucratic overhead that we have created, which may be necessary for a test environment, but it sure had better now be for broader implementation. I mean, it’s frankly bonkers that we have got to go to such huge lengths to get practices enrolled and get GPs enrolled and get patients enrolled (MG4 Round 2) |
| **Program and funding time limited**   - *Not enough time to see results* - *Cost benefit is a longer term outcome* - *Closing as awareness is building* - *Difficult keeping staff* - *Staff invested in success of program feel responsible* - *Uncertainty around future beyond pilot* - *Concern about WSICP enduring affects staff commitment* - *Limited ongoing funding has been secured* | | | *I’m worried that the funding stops and all of this is a waste of time. I don’t think you can see results in two years, I don’t know that you can even see results in five years, but I think it needs long-term investment in an initiative like this that focuses on outpatients and keeping patients well in the community (AH6 Round 1)  *The key thing is whether that investment is continued, because it takes a long time for things to get going, as you know, and this was really recently considered as a true four year project, but really it’s only just getting started. (MG1 Round 1)  *the cost benefit in terms of the patient getting better care, avoiding hospital, getting them out of hospital quicker and getting them out of emergency quicker. But I think these things take time and the time frame is quite limited of this program (HS11 Round 1)  *it will be good to actually know that there will be some, sort of, long term plan for this. Because it is hard to hold onto good people when there’s a time restriction there. We’re having a high turnover of our educators - it’s very hard (HS5 Round 1 and 2)  *it’s going into mid next year [2017], that makes it very difficult people are worried about what’s going to happen after that, a lot of turnover of staff– then you have to start again and retrain people and fill jobs (HS3 Round 2)  *We know we're in this for a change that has to happen and be sustained and if you set it up as a demonstrator what happens when the money runs out, what happens to all the people that you've brought on and are starting to catch on and how can you employ people and give them a 12 month contract and expect them to have their heart in the system? So I think that the system is flawed because it is a project (HS8/MG7 Round 1)  *I’ve seen so many different programs come out from the Ministry of Health and then it just fades and dies. And I think initially that may have also led to some of the resistance internally, with inside the hospital because doctors get a bit jaded (HN5 Round 2)  *There is continued funding available, it may not be of exactly the same quantum and there may also be some new investments available (MG3 Round 2) |
| **Time needed to establish program**   - *Embedding program in system* - *Program developing over time* - *Working through difficulties* - *Expectations of worthwhile outcomes including new ways of working* | | | *the program’s still very, very new, everyone, like I said, it’s a very new program, we’re trying to figure out the referral pathways and where everyone sits within it (AH2 Round 1)  *we’re at a point that we’ve got a reasonable way; happier now than I was six months ago in terms of how much we’ve delivered. I think we’ve finally got our team and our models of care in place when we’re at that point where we’re testing them and seeing whether they work, and there’s a number of frustrations there that haven’t been worked through yet (HS1 Round 1)  *I don’t see a lot of GPs actively ringing the RASS service despite our promotion, sometimes, unfortunately on the negative side, we’ve had a few bad experiences as well in ringing RASS like no one answered the phone call despite leaving voice messages, no-ones contact them back. It’s improving … (CF3 Round 2)  *we had so many problems with our computers we could not upload it and actually get patients in. For about a year they [WentWest] were working on our computers trying to make it work and it wasn’t until either December or January this year [2017] that finally the links are now working and we can upload the metrics and get patients on (PN7 Round 2)  *if the GP hotline doesn’t ring then I’m not going to be able to do anything, and I knew that setting up the breathlessness clinic was just going to take time and, again, it’s going to be a slow burn.(HS2/MG9 Round 1)  * It’s just unfortunately a lot of these really good ideas take a lot longer than expected but that doesn’t mean that it’s not going to be worthwhile in the end. It’s just taking us longer to get there. (GP5 Round 1)  *This is certainly a very good step, it’s one year down the track in terms of the interview now - we’re still definitely in the infancy of it. (HS10 Round 2)  *I don't think we can go back to old ways of doing things– it’s just like we’ve still got training wheels on (MG6 Round 1) |
| **Challenges and time required for behaviour change**   - *Time required to build awareness and embed new GP behaviours* - *New hospital based approaches require culture change* - *Some GP attitudes a barrier to engagement* - *Education of patients in accessing services more effectively* - *Noticeable changes in how people think about providing care needs to translate in practices* | | | * we have to allow the time to get this message out to the GPs, allow time for changing behaviour. Which takes time. I think, we’re not even a year into this and I think we’re trying to change a system that’s been in place for a very, very long time. (HS3 Round 1)  *the other problem I think is the GPs fall-back position of, they’re unwell, they go to ED. (HN5 Round 2)  *they have some confidence in us, they probably feel more comfortable referring more patients to us. I think we are changing the GPs practice behaviour, in a way (HS7 Round 2)  * I think it takes time for a GP to understand how to use it, because some of the calls have not been appropriate. (MG1 Round 1)  *We’ve got some doctors, particularly the young ones, who, yep, we can do that, but there are doctors who’ve been around quite a while and are a little bit resistant to big changes, have really struggled and one doctor hasn’t come to any of the sessions that have been put on to learn about patient integrated care at all, he’s just completely uninterested (PN3 Round 1)  *within the patient population, their lived experience is “I get sick, I come to hospital, I get admitted so I must need to be in hospital”, and there's all this sort of sick role, you can’t change that for the current group of patients. You have to wait for them to shuffle off the mortal coil essentially and re-educate a whole new group of patients, and that’s just going to take time (HS2 MG9 Round 1)  *We're seeing a change in people's thinking. I think it's more embedded in people's thinking. I don't know that it's embedded in practice yet. But I'm not surprised. These things take time (MG6 Round 2) |
| **Time required for chronic disease outcomes**   - *Already seeing an early impact to chronic conditions* - *Challenges in dealing with comorbidity* | | | *we’re focusing on chronic disease, so chronic disease, in its very nature, is not something that changes quickly or that you see an impact quickly and so I think the fact that we’re actually seeing something so soon does suggest that this is going to be beneficial in the longer run. But it needs time. It really does (HS3 Round 1)  * I think that it’s very early days but, we’re starting to show some improvement in primary outcome measures which is the breathlessness management (AH4 Round 2)  *COPD patients have a lot of emotional issues. So focussing on that, is really important, to reduce admissions to hospital …when people get breathless the first thing they want to do is call ambulance, so teaching them how to manage their breathlessness and their COPD at home; is so important. (HN3 Round 2) |
| **Value Add** | | | |
| ***Building Capacity/ Education/ Upskilling*** | | | |
| Patient upskilling | | |  |
| **Advocacy and patient empowerment**   - *Patients are making their own decisions* - *Information empowers patients and provides choices* - *Patients better able to identify their care providers* - *Carers prepare before meeting doctor* - *HCPs collaborate to support patients in working on gaps in their care* - *Educating patients builds awareness and understanding and they feel supported by CFs* | | | *So yeah, I make my own decisions on my health, taking those kind of medicines. So I’m aware of what I’m doing. Because I don’t want to be hospitalised (PC2 Round 1)  *If I'm not feeling good or whatever I'll just go and do it. I don't need a committee to get something done. I'll just go and get it done (PC4 Round 2)  *They’re a bit more proactive in coming in. I’m seeing more patients, which is good, and not just because they’re unwell, it’s because they need something checked as part of diabetes, like blood care and stuff like that (PN3 Round 2)  *– he suddenly reached the point where he actually wanted to make a difference, wanted to make a change and so the RASS clinic was perfect. He was so determined. (GP5 Round 2)  * He’s able to empower himself, he’s learned what to look for, in particular heart failure, how to manage his weight, how to manage his salt intake. What to do with his diuretics. At a ground level he knows what to do now, and so he does that and whenever something goes wrong he’ll see the GP. (HS10 Round 1)  *It’s very easy to forget what you want to ask when you actually get to see the doctor so I tend to write it down. I like to take my book with me with the readings and say, “Why do you think that happened there?” And try to get answers. (PC22 Round 2)  * we’re trying to identify where there might be gaps in care or where things might be falling down, and then really … supporting the patients to work on those gaps themselves … So getting them to go to their specialist and say, “Look, I know you’ve look at this but I'm really concerned about this, can we have a discussion about it?” (AH3 Round 1)  *One of the main barriers is patients knowing who their specialist is, and you say to them, “Okay, we need to write down all of your health team,” “Oh, I see someone who’s my cardiologist, I just can't remember his name.” But, I think, as we’re giving the patients more and more training … we’re assisting them in keeping their own records more effectively, so that they’re able to name those people. (AH3 Round 1)  * through the time and education spent on them they are able to take matters into their own hands a little bit more in terms of choosing better foods, or maybe for their exercising, or they're doing those lifestyle changes that is going to help impact their health definitely, because they feel like they understand why they're doing it a little bit more, it's not just do this, do that, now there is a reason behind it(GP6 Round 1)  *after the last case conference I had a few patients who came back to follow up with me and they felt a lot, I guess empowered was the word, with the information they had. And also other resources that they can access at home when they had instead of, just running off to the doctor (PN4 Round 2)  *sometimes patients tell me when I have been to the GP with them for my first visit, the GP has explained in a lot more in detail about their condition, rather than when then patient goes alone so I think sometimes just having an advocate, having an educator or having someone with them, it has a big impact on definitely service navigation and the understanding of their chronic disease (CF3 Round 2) |
| **Collaboration in decision making**   - *Patients and GPs work together* - *Change from medical model of care engages patients in decisions* - *Patients are proactive in their treatment* - *Doctors check understanding with patients* | | | *they leave it up to me, they don’t come across with the decision to what I should do, so, ultimately, if we make me feel better I’ve got to listen to them and put it into practice. (PC14 Round 2)  * So I still had some of that left, so I said to the GP, “Oh, look, I’m thinking – I’m just about out of the one called duo and what if I go back on the metformin and see how I go and I’ll just keep measuring my sugars and see how we go?” And he said, “Yeah, that’s a good idea.” (PC3 Round 1)  * I think that if you’re working in a hospital setting… what happens with the patient is decided by the medical staff, and the doctors, whereas this is more about the patients here, and they have a hand in deciding what’s happening to them, or with them, and it’s more having them as the focus of the care. So it’s a shift away a little bit from the medical model… (AH4 Round 1)  *it empowers them, because they actually have a say now in how they are treated. They show keenness in motivating themselves to get well, they actually get much more from the program than just waiting for something to happen to them (HN6 Round 1)  *being a patient, you’ve got to communicate properly with them, you know? If you're unaware of what they're saying, ask them to explain it in layman’s terms. I've learnt that now. I've learned not to just accept (PC2 Round 2)  *when I’m in hospital. They come up and ask you “is there any questions or do you understand this”? (PC12 Round 1) |
| **Learning about the condition and self-management**   - *Providing health care that has a strong educational focus* - *Case conferencing improves self-management for diabetes and reinforces the message* - *Patients learn about their illness and how to manage but not all carers receive information they need* - *Some carers and patients self-educate* - *Action Plan has step by step instructions for patient* - *Key role of allied health and CFs in patient education including in the home* - *Education and self-management reduces re-admission rates* - *Prevention and follow-up keeps patients out of hospital* - *Improvements in patient health and satisfaction noticed at primary care practices and the hospital* - *Patients keep log books to actively self-monitor* - *Educational leaflets and brochures, and websites and telephone services also help patients self-manage their illness* | | | *They’re brilliant, they explained what will happen, and how to deal with it (PC14 Round 2)  * There's patients have attended diabetic for educating, education sessions while in the program also respiratory type of education. Yeah. I must say it has improved. (GP1 Round 1)  *Oh that [case conference] was extremely valuable, highly valuable for everyone. I think every patient that came through there were very good suggestions - and all of them were taken on board. And all the patients who were involved in that have better outcomes (GP9 Round 2)  *the diabetes case conference, that’s helped patients self-manage and I’ve definitely seen that a number of times for the patients on the integrated care program…. (GP5 Round 2)  *I’ve been saying the same thing to the patient and achieving nothing, but when the team comes, the [patient] goes, okay, listens, this is the message, and just does it but it’s actually good having someone else with the authority, reinforcing it, I think, makes a lot of difference (GP14 Round 2)  * I’ve learnt a lot more about my heart problem and I have issues, what, with the lung and very bad infection of the lung and that and that took about nine months to get over, but, yes, I have learnt a lot from it. (PC7 Round 1)  * some patients have gone to the COPD clinic and they’ve taught them or they’ve gone through their medication use and what medications do and how to actually administer them properly, so that’s been helpful for managing their own condition.(HS9 Round 1)  *We're going over everything; you know weight, fluid intakes. Keep an eye on the weight (PC4 Round 2)  * was rather hard being his carer…to find out information. “Oh who are you? I’m his carer”. Even now, we’ve been in Westmead, Blacktown and Mount Druitt in the last couple of months, and I walk away shaking my head. (PC19 Round 2)  *I’ve done all the, like research online by myself, so, I think, I've learnt a bit through there (PC15 round 2)  *my father calls me Dr Google. I said, “Yeah, well it's my body.” But I'm not stupid to believe everything I read. (PC8 Round 2)  * we give them an action plan which is when I get these particular symptoms this is what I do. I start taking more of my puffer and if that doesn’t work then we’ve got Step 2 which I start taking this prednisone and/or antibiotics and I go and see my GP. And for some patients they really get it and they do it. (HS2 MG9 Round 1)  *the dieticians and educators are some of the key players in enabling patients, because they often have the advice for day-to-day practical matters (HS4 Round 1)  *I had a patient who’s a newly diagnosed CCF and [CF] is brilliant, I was sitting here listening to her educate the patient on his newly diagnosed disease, she really is very, very good. The family were really pleased and I could sense that they felt quite comfortable with her knowing that she would also be a part of the team if they were in hospital or sent down to the RASS clinic and I think it was very well done. (PN3 Round 1)  *most of them do feel more confident at the end of the program, in managing their breathlessness, and less anxious about being breathless (AH5 Round 2)  * when it’s their home environment, they’re far more open, to what education you’re providing, and they can think in their own environment about how what you’re saying applies to their meal preparation, their shopping. (AH6 Round 2)  *we’ve slightly decreased the readmission rate, so I suppose that’s something, as in they’re weighing themselves regularly, they’re watching their fluid restriction, they’re taking their medication. Maybe we’ve got some people to stop smoking (HN2 Round 1)  *we're doing a lot more preventative things and keeping in touch with the patient, it does improve their compliance with treatment and keeps them out of hospital. It has benefited the patients and a lot of them are keen to continue (GP12 Round 2)  *we've seen improvements directly through HbA1c improvements and day-to-day glucose readings. So, yeah, they've been some very clear improvements and patients have engaged well (HS4 Round 2)  *when they have eventually come to hospital they’ll tell us, “But look I’ve been watching my weight,” and this and that. So they flag the things that have been flagged and they keep their own log books and they come back in, provide the log books. I think they certainly have learned, how to, watch for deterioration, what to do if - and we’ve developed patient’s plans with them and it’s certainly given them that sense of ownership about their own health (HS10 Round 1)  *it definitely improves, because my patients have a really good education from the clinic. They are very happy. (GP3 Round 1)  *the patient does see a specialist as well but the heart failure CNC has been able to kind of give them some other education about their condition as well as monitoring and any other suggestions that may be able to assist the patient in that sense (GP4 Round 1)  *there’s a lot of educational leaflets and brochures and education provided in terms of the disease, puffers and smoking cessation, and so forth (HS11 Round 1)  *They have a Better Health website, which is good; we can give them the resources available, can easily print out what’s available, and then hand it over to them. Even the GPs can easily do that as well. It’s, basically, a source of library for everybody else out there in the community (CF4 Round 2)  *How to control – if I’m having a panic attack. If I’m having an anxiety attack, which has been – they’ve been so helpful up there. And, just the general breathing part of it. (PC11 Round 1) |
| **Navigating the health system**   - *Patient hotline gives patient a central point of call* - *Patients can contact CFs but not all have easy access* - *Some patients prefer to go through GP* - *System provides familiarity to patients* - *Patients also followed by clerical staff- impacts DNA rate* - *Patients and carers know who to call and when to call-awareness of services* - *WSICP has prompted HCPs to think about patient navigation but some patients are still highly dependent on their GP* | | | * I certainly see patients ring up on the hotline, so I think that’s - they feel that that’s better, and our sort of bits and pieces feedback from them has been that they are happier with their understanding of their condition and who they need to talk to.(HS1 Round 1)  * I think, the patients are, like I said earlier, feeling like they’ve got a central point of contact and that they’re not lost in a sea of people that are trying to help them that are not connected and they don’t know where to go, and, I think, they’re feeling like they know where to go to get the help that they require.(AH3 Round 1)  * I think they appreciate that that there is a care facilitator as well with a bit of a one stop kind of shop if they have got questions or problems, help them navigate the system (GP6 Round 2)  * Sometimes they’re not sure whether to call – we’ve given them online and mobile numbers but some of them … have to rely on a landline and they might be not have access …Also not having IT knowledge (CF4 Round 2)  * they still don’t see us at the point of contact, they would much rather do their GP and then get the GP to call us, which works well as well (CF2 Round 2)  *… a familiar face to the patients that come and see quite frequently in the hospital setting (HS10 Round 1)  *they get a number to call, they can just pick up the phone and ring the nurse or pick up the phone and ring the doctor and that’s kind of all they need. They’ve got so many other things going on, so the fact that they can navigate that or go to the GP, get the GP to call us, I think that is a big plus, the navigation aspect (HS11 Round 1)  *I think it’s made things a bit easier for them… because we’ve got a clerical person, they’ve been able to follow them up…that’s had a huge impact on the do not attend rate (HN2 Round 1)  *I think it’s made things a bit easier for them… because we’ve got a clerical person, they’ve been able to follow them up…that’s had a huge impact on the do not attend rate (HN2 Round 1)  *Now I know who to call and when to if I need them (PC7 Round 1)  * Good to know that, there’s things out there that I can get on to if I need to find out anything more, which makes it easier for me (PC16 Round 2)  *I’ve said to a number of patients, if you start to put on more than two kilos, if you’re not able to do the same sort of activities as you were, if you’re getting more short of breath, call your GP, try and get an appointment.” They go, “He only works two days a week.” “Okay, you call us and we’ll arrange a clinic appointment.” But I’m always going to stress that they need to call their GP first because they’re the primary person responsible for their care (HN5 Round 1)  * …if you're away and something goes wrong, they know who to call apart from us. (GP6 Round 1)  * And, so, integrated care has helped me think about the patient’s navigation through the system and how all the little bits link up together.(MG1 round 1)  * the patient is very dependent on the GP, or the practice nurse on making phone calls, so they come to me and then hopefully I’ll know where and who to call (GP8 round 2) |
| Training and Education of practitioners | | |  |
| **GPs and practice staff**   - *Hospital specialists from the WSICP are visiting general practices and providing education in the program’s three areas of chronic illness* - *A multidisciplinary approach to education* - *GPs prefer case based approach* - *Education increases general practice awareness which may increase referral* - *Hospital specialists learn about general practice needs* - *Hospital allied health providing education in the community and building relationships* - *Hospital admissions are reduced* - *Need for education about integrated care* - *General practice staff learning and building skills- satisfaction* - *Case conferencing builds GP confidence and relationships* - *New practice skills translate to patient self-management* - *Education workshops are not always taken up well by GPs* | | | * Because we're looking more at the practice nurses and that sort of thing and there is education going on for them in the three disciplines, diabetes and respiratory, we've already done that, and we starts ours in the middle of June, so they'll get four sessions on heart failure.(HN4 Round 1)  * respiratory were saying maybe we could come out and show you how to do spirometry and I think that is part of a similar thing but I have tried to encourage them that it actually is better if we have the patient and talk about particular patients because that’s actually means I learn a lot but yes it is part of the skilling up process (GP5 Round 1)  *three times last year an endocrinologist came to my rooms at three different locations seeing my diabetic patients, they brought their diabetic educator, and we lined up my poorly controlled diabetics to see them with us. (GP8 Round 2)  * the conferencing we have done has mainly been with or without the patient present, and then with the care facilitator and with the public community nurse, with our pharmacist, the dietician and allied health professionals (GP6 Round 2)  *My work in diabetes, which is also aligned with the Integrated Health Demonstrator Project, is about building the capacity of general practice to better manage diabetes and the way that we have been doing that is with a series of interventions that one of those is case conferencing (HS8 MG7 Round 1)  *we’re coming out to extending –but also explaining and educating and that also, maybe in some sense, could actually end in referrals (HS5 Round 1)  * going out to GP practices and doing some teaching has been incredibly eye-opening, I’ve got a much better understanding of what it is my GP colleagues want and need. (HS2 MG9 Round 1)  *Based on that feedback that I got from dieticians within the community, and also our OT and psychologist got from those relevant disciplines in the community, we decided to run an education evening for allied health in the community, just to share what we’re doing, and what messages we think are worthwhile reinforcing in the patient group, so that was a really good opportunity to network outside of the hospital (AH6 Round 2)  *Conferences have been really good. They've been good in terms of formulating the plan and avoiding unnecessary referrals (GP2 Round 2)  *education as far as GPs, yes, there has been some education done but it probably needs to be a lot better from WentWest sort of process, because I don’t think the GPs actually know that it’s there half the time (HN2 Round 1)  *I am learning from sitting down with [CF] [laugh], she’s been - seriously she’s awesome, I really like her she’s good at her job and she knows her stuff and I have learnt and I’m also coming from a paediatric background so all this adult stuff is pretty good for me, so I’m learning a lot (PN3 Round 1)  * I think understanding of the management of the patient has improved, so I think overall it's starting to improve education as well of managing the patient (GP6 Round 1)  *It’s been fantastic [case conferencing]. The treatments from the GPs have increased. They’re more confident to changing medication and up medications. And the patients seem to like it as well because they prefer to come to their GP than go to a specialist (PN3 Round 2)  *they upskill GPs. I still feel anxious about starting people on insulin but I am able to do that now, whereas before I wouldn’t have felt comfortable doing that. (GP5 Round 2)  * our nurse has been educated in diabetes, it really improves her knowledge. She’s meeting up with patients. The fact that she is more educated and knowledgeable about diabetes is also then transferring to our patients in helping them self-manage as well (GP5 Round 2)  * we go out to GP practices, but we’ve also put on a couple of education nights for GPs which have been pretty underwhelming, to be honest. We get less than 10 GPs, for our education night. We get some general practice nurses and some other practice managers and things, but in terms of educating GPs that’s been a monumental waste of my time. Because they just won’t turn up, and I put on free meals for them, it’s been in different venues (HS2 MG9 Round 2) |
| **Hospital and specialist**   - *Clinical and integrated care educational initiatives in the hospital* - *Learning and teaching are reciprocal* - *Working with others facilitates learning* - *Adapting theoretical knowledge practically* - *Learning to manage comorbidity* | | | * We’ve had guest lectures on COPD and other sort of Breathlessness Clinics, we’ve had library education, we’ve had in-house education from registrars about COPD in terms of what they look at and FEV1 and all those sort of things that some health psychologists aren’t so up with. So it has really been educational for various different health professions, different perspectives, so OT and dietetics and so on, so everyone showing their piece of the pie (AH5 Round 1)  *There are a number of seminars, more so planning meetings and to speak about the intention of the integrated care program. (HS5 Round 1)  *for our own training purposes, and we speak on a regular basis to those in the other teams such as cardiology and also respiratory, so that’s great stuff (HS4 Round 1)  * I’ve been attending the meetings every six weeks, we have these operational meetings and I get to learn about, the integrated care how its developing and all of the challenges and how we are working towards, mitigating those challenges (HS6 Round 2)  * I work with an amazing team. And we always do education to try and make sure that each member can teach the other member of the team something new about – from their speciality. And I had an amazing consultant, and CNC’s that mentor me (HN3 Round 2)  * I think, the emphasis on education for those sorts of areas has been really huge, really valuable, really steep learning curve, but, yeah, I’ve probably learnt more in the last three or four months than I have for a very long time. So, just that gain of knowledge from working so closely with them. (AH4 Round 1)  * I’m actually more au fait with how to manage foot wounds, the influence of off-loading, the kind of shoes they have to wear, the important of blood pressure control. And we’ve also moved into the area of anxiety and psychology and mood, because that’s really important (HS5 Round 2) |
| ***Changes in Practice*** | | | |
| **Collaboration/ communication between community and hospital**   - *Cooperation between Primary and Tertiary sectors to integrate care* - *Hospital connects with external providers* - *Hospital staff connecting better with GPs – and learning of their needs* - *GPs use WSICP rather than sending to ED* - *Hospital allied health now better connected with community allied* - *Feedback from GP to hospital is not always direct or frequent though possibly changing in second round* - *Some GPs better informed when patients are admitted* - *More immediate communication from hospital to GP* - *Hospital Follow up post discharge useful* - *Care facilitator is pivotal in hospital and General practice communication and in connecting strongly with community services* - *GP staff more aware of services* - *Referral to specialists easier* - *Limited number of patients in WSICP in early days* - *Education at general practices establishes stronger links with hospital* - *Practice managers visit hospital for education* - *Patients and carers note greater collaboration between GP and hospital and improved feedback* - *Carers receive feedback* | | | * Well, the most positive factor, I think, is the spirit of cooperation between primary care and hospital care. And, so, those who are involved in integrated care, whether they come from primary care or from the hospital end, have been very enthusiastic and keen to have integration of care. (MG1 Round 1)  *We have a contact with external imaging services that are able to accommodate, the level of investigations we require, in terms of the volume.(HS6 Round 2)  * As a result of the program, there are - for my service there has actually been better communications between some of the GPs and us, and I'm finding I'm getting more responses, or we're getting more responses from the cardiologists. We've had a more formal letter going out with the care plan on it to all cardiologists that meets their needs, so they're more aware of what we do.(HS9 Round 1)  Historically, if GPs were unsure of these patients, they would just say, “Go to emergency,” but if they have something to fall back on, again, that’s helping out the community in terms of building a bridge between primary care and the specialists, respiratory and cardiology and endocrinology.(HS6 Round 1)  * Yeah, look, I’ve been in contact with community dieticians that I haven’t been in contact with before and I think that will have benefits in both of my roles.(AH6 Round 1)  * I feel like I can how have the capacity more to contact GPs and write them letters and say, well I’m happy to keep seeing the patient here, however could be beneficial for them to see the private dietician (AH2 Round 1)  *my role is to arrange some long-term follow up - actually involves liaising with their GP to organise for them to see their GP on a regular basis for a long-term review (HS5 Round 1)  *The doctors from the hospital are more into calling us for more information, I think, there’s no hesitancy to ring us if they need help (GP8 Round 2)  *We do seem a little bit disjointed, I’ve had GP feedback but not from the GP's, it's come via a third, fourth, fifth person and it would be much better that if there was an issue that they discussed it with me. (HN5 Round 1)  *We see there are actually more GPs contacting the service with regards to referring their patients to our care. There are more doctors being talked to by the care facilitators in regards to how the services are being done (HN6 Round 2)  * I’ve worked on getting quite a good communication going between the community nurses and the heart failure service, so that’s continuing. GP wise sometimes we get something, a lot of the times we don’t; we’re sending them everything and hearing nothing back (HN4 Round 2)  * the feedback or the communication has been much better. I'm seeing changes or I'm learning about patients being in hospital much sooner. And therefore I'm able to follow up those patients a lot sooner as well. (GP9 Round 2)  *the patient that's in the Integrated Care Program, they go to hospital, I get notified, which is excellent. It would be good if I had that with all my patients. (GP2 Round 2)  * The only thing we are kept out of the loop lately is when our patients are admitted to hospital (PN3 Round 2)  *From a GP point of view I still feel that maybe the doctors in the hospital aren't as well aware of the program requirements (GP4 Round 2)  *the relationships between general practice and the specialists are greatly improved and therefore I think you are able to actually manage more disease better and earlier in general practice (HS8 MG8 Round 2)  *while I might call some of the GPs and have a chat to them about the patient if they’re quite a difficult patient or quite complicated, I still haven’t met that GP. There’s a professional relationship but it would be nice to know who you're talking to (HN2 Round 1)  *… I call the GP from the clinic and say, "I have your patient here. We're going to do this." So it's not just a letter. (HS1 MG7 Round 1)  *I feel that the discharge summaries have been coming out really promptly and really good summaries – good communication from the hospital about that (GP5 Round 2)  *the main person it changed with is the care facilitator. I think that is the person who is having the most contacts with us still face-to-face or on the phone or anything like that. (GP6 Round 1)  *I think it’s easier to refer to the specialist than before (GP3 Round 1)  * There is a shift in the thinking with the hospital - between the hospital and the GP. There's more communication with the rapid access clinic and things like that. It was very hard for us, previously to try and talk to someone and get them admitted but now it's a lot easier. (GP12 Round 2)  *I only have a few patients who are linked up with the integrated care program. So, for example, that heart failure CNC, I got correspondence back on her I think within a week or so (GP4 Round 1)  * we have multiple talks with the GPs and even visiting practices to talk to GPs about education; it’s opened up those links as well and we can’t forget also communicating with the patient because obviously they have a point of contact, but the feedback has been very positive for patients, to have a point of contact (HS11 Round 1)  * linking the specialists in with the GPs that are looking after their patients as well, so it's definitely helping the communication side of things, and it's definitely helping with access which is a really big part of improving the care of the patient. (GP6 Round 1)  *recently we had little visits from practice managers. They came in to review Integrated Care Clinic to see what went on, so that they could better understand what happens with us and they could relay that back to the GPs (HS4 Round 2)  *I think there maybe more contact with Dr [GP] from the hospital because of it [WSICP] (PC9 Round 1)  *We are on the same track. The psychologists, the doctors, the care plan clarified – hopefully you get to understand drugs, how to manage correctly, so they are good. It’s also, like an assurance for me that I’m doing it right. As a carer (PC18 Round 2) |
| **Collaboration/ communication in the hospital**   - *Greater collaboration and communication across disciplines to attend to patient comorbidities* - *Collaboration includes multidisciplinary team care* - *Cross hospital collaboration improved* - *Rapid access clinics facilitate within hospital collaboration* - *Collaboration is not always equal across disciplines sometimes due to location* - *CFs relationship with management more effective* | | | *it’s given them more access to each other. We were working in siloes before almost didn’t know the other was there (HN4 Round 2)  * So many of the patients are shared anyway, you know, they sort of bounce back and forth from the normal heart failure program, integrated care, when they have deteriorations and so for, so, yeah, we work as a team.(HS9 Round 1)  * we try and link them on the Friday morning if they’re there seeing their cardiology, then, yeah, we get them sent to us, and the same with respiratory, there’s been a couple of referrals recently from them into our clinic. (HN1 Round 1)  *with patients that require, for example, endocrine and cardiac input, we will try and put them into our combined clinics on a Friday (HS1 MG7 Round 2)  *my Thursday morning foot service, there is also the infection disease department because they’re also vascular, so the actual surgeon is there, and quite a lot of foot doctors as well – podiatrists – we’re engaged with them a lot more than we were, which is fantastic (HS5 Round 2)  * I know we’re all trying to work to the same thing, and there’s care coordinators out there trying to recruit the GPs. But, I think, the three chosen areas, as it were, I don’t think we’re talking that well together (HN1 Round 1)  * Diabetes and cardiology want to work together to see patients with comorbidities so that’s great.(MG2 Round 1)  * Also we’re now aware of the groups in Blacktown as well, so I think that there definitely has been improvement in that (HS1 Round 1)  * the CNC nurse and doctor work well together, they’re connected, and by involving emergency and respiratory even at Mt Druitt emergency to give some talks because we get admissions from there to Blacktown Hospital, and also they’re getting better with other specialities, with cardiology and diabetes working together with them and having joint meetings with them (HS11 Round 1)  *we meet once a week following clinic when we go through every patient and who has been involved and what the outcomes were and what to do next. So for the whole 8-week period, they get reviewed each week. (AH4 Round 2)  *being able to access the rapid access clinics for diabetes and cardiology has been really useful because I’ll see a patient and I’ll go, actually their sugars are a bit all over the place and I’m a bit worried. And before I wouldn’t have known what to do that but now I know I can pick up the phone and call diabetes rapid access (HS2 MG9 Round 1)  *the collaboration needs to improve, certainly in this program there's closer collaboration between respiratory and cardiology, very little with endocrine and I think that’s something that we have to work on but I’m not really sure how to be honest because they’re quite spread away. (HN5 Round 1)  *we’ve had a couple of patients that we’ve had multidisciplinary approaches with, with the endocrine, not so much with respiratory here…We have a multidisciplinary day every Friday morning if required (HN2 Round 1)  *the problem is in respiratory where it is so isolated from where cardiology and diabetes are. So we’re up on Level 5, while they are on the ground floor…they might have interaction with each other, compared to respiratory (HN3 Round 2)  *the team has become more collaborative, within our own management team we’re all working together this year so they are best to understand our frustrations and try and translate that into solutions for us, that’s changed (CF2 Round 2) |
| **Team based care**   - *Multidisciplinary team includes allied health* - *Linking GP and Hospital for complex patients* - *Linking to private specialists* - *Team based care addresses patient needs and includes home visits* - *Case conferencing facilitates team work* - *Increased GP capacity to do more on site helps carers* | | | * I guess, there’s another thing I’ve noticed, is there’s – when you’re endocrine, then you’ve got your chronic heart failure and then the COPD, the respiratory team, and I find that, yes, we are integrated care (HN3 Round 1)  *meeting with all the – other specialties like cardiac and respiratory, it’s really good. You can see then what they’re doing together, like we work together (AH1 Round 1)  * we're lucky that we have got such a good team environment here, so our allied health are onsite and easy to communicate with, you can do joint consults, you can team up which makes the communicating side of things a lot easier… it really helps to optimise the patient care and share the care around (GP6 Round 2)  * here at the moment we have a dietician and an educator – we have our clinicians and the registrar as well who also works with us (HS5 Round 1)  * In terms of Allied health, I would say – it has increased, I mean I go to different case conferences and different practices where the patient’s reviewed with the exercise physiologist and we’re all around the one table and we talk about different patients (CF3 Round 2)  *the multidisciplinary team we’ve created for the breathlessness clinic has been really useful and it’s really interesting because we’ve got OTs, the dietician, a physiotherapist, a nurse and a psychologist on that team (HS2 MG9 Round 1)  *we work alongside allied health, often sharing the room and doing something and they’re doing something, we’re all working together rather than the educator being separate to me(HS4 Round 1)  * they’ve been able to employ the dietician and educators and I guess before those patients wouldn’t be touched, but now we are getting through more and more people.(AH2 Round 1)  *I never had all this dieticians and things like that but now all of a sudden everybody’s getting involved (PC10 Round 1)  * The OT goes in and enters their home environment to try and see if they can improve and make life easier and happier for them. And then if we think they have problems with their level of anxiety or depression, then we refer them to our clinical psychologist (HN3 Round 2)  *We see dieticians down there and there’s someone there from the chemist, like to help you with your medication… since he’s come home there’s always someone ringing me up or coming out (PC19 round 2)  * with the case conference and I think it is a good thing, because we can see everyone’s input on it and we can work together as a team to better manage this patient (CF4 Round 2) |
| **Keeping people well - focus on preventative health (and seeing patients earlier)**   - *Organisational direction to prevent illness* - *Patients no longer need multiple hospital visits* - *Some patients visited at home when they cannot attend hospital* - *Patients seen early in disease trajectory* - *Short term interventions improve patient outcomes* - *Rapid access and stabilisation is preventing complications and admissions* - *Early intervention allows a focus on self-management* - *Regular monitoring is noted positively by patients and prevents hospital admission* - *Keeping people well extends beyond the medical to the social domain* | | | * so, at an organisational level, we’re looking at how we better manage health in Western Sydney and how we keep people well as opposed to how we treat people that are sick. (MG2 Round 1)  * before the Integrated Care Program Mum was constantly either getting admitted into hospital, like I said before, so they would treat or fix one issue and then she’d come out of hospital and she had something else wrong… The medication was constantly being changed, whereas now the Integrated Care Program over the last six months I think she’s been into hospital twice… she goes for two months with her blood pressure and her sugar and everything on track. (PC1 Round 1)  *they're feeling a little bit sick. They cannot attend the appointment because they are feeling unwell, in which case we're just coordinating it with the CNC, who is more than happy to do home visits (HN6 Round 2)  * we’re going out and doing home visits, it’s also that they’re quite frail, they’re very breathless, they’re often carting around oxygen tanks, so a lot of people are really hesitant with respiratory conditions that come to the hospital environment, because they might pick up a bug and get sick and land up in hospital (AH6 Round 2)  * This provides a service where we see these patients very early on within most of the patients nowadays we’re seeing within 48 hours.(HS6 Round 1)  *we picked up in a week there was two patients which we didn’t even think were going to be an issue, ended up going for emergency by-pass. So, definitely prevented a heart attack and definitely prevented them dying (HN2 Round 2)  * huge research that shows diet and exercise is better than anything else. So I guess our educators here, it has – hopefully you’d think in the community it’s made a difference and will prevent admissions, will delay complications of onset of diabetes (AH2 Round 1)  *we’re picking up changes earlier and keeping on them, it does help them to self-manage a bit better. (HN4 Round 2)  *For me just ongoing monitoring is the main thing. …all of it’s been positive in the way of the care I’ve gotten once something’s happened. (PC4 Round 1)  *we're doing a lot more preventative things and keeping in touch with the patient, it does improve their compliance with treatment and hopefully keep them out of hospital (GP12 Round 2)  * urban design, transport, food supply and physical activity, and then identifying people at risk of, say, chronic disease as an example and then working with primary care as they interface with the health system to keep them healthy and keep them well (HS8 MG7 Round 1) |
| **Patient centredness**   - *Patients receive better communication* - *Patient care individualised* - *One–on-one care* - *Patients know they are the focus* - *Doing what is important to the patient* - *Patients feel heard* - *WSICP changes how care can be provided in the hospital* | | | * Oh, yes, I believe it has, I find that the people are more forthcoming and at least you can stay and talk to them and you’ll get an answer.(PC7 Round 1)  * they really feel a bit special, a little bit like, hey, this is the first time that I really have been looked after, in a way that they can understand, in a way that they really seem to appreciate and can engage with (AH6 Round 2)  * we were all giving him information, but, I think, he was quite empowered, because he knew we were all there doing the different things, but he was still the centre of what was happening (AH2 Round 1)  * might see that them not being able to get a shower is really very important, but they may not. So, therefore, I don’t work on that. I work on other stuff. Making a cup of tea, walking to bingo, or whatever, it has – into the garden. Sitting in the garden, whatever it is, so it is becoming more focused on that. So what they want to achieve (AH4 Round 1)  *The cardio, when stuff has happened, he fits me in ahead of schedule on things, so everyone’s been good. They actually get it done. They know I'm not a hypochondriac, so if I say something is wrong they listen (PC4 Round 1)  *when you were working in a large hospital, with 50 patients to manage, there was no luxury - to do good care was a luxury. It was, logistically, difficult. But the Integrated Care Program is so amazing that we have - because of the patient focus, everything we do is so mindful, that it is all about our patients (HS7 Round 2) |
| **Holistic Care**   - *Addressing complexity* - *Comorbidity especially psychological needs addressed through multidisciplinary patient centred care including in the home* - *Time efficiency for those with multiple needs* | | | * some of the more complicated patients… have received more intensive care. (GP1 Round 1)  * From a psychology perspective, it’s a big difference, because psychology is not often included in a lot of medical conditions whereas a lot of people with medical conditions have pre-morbid anxiety and depression and it’s a big cycle. I think treating one helps the other.(AH5 Round 1)  * the fact that they can get a lot of different services rolled into one will be attractive for patients that they can not only have their symptoms addressed by the doctor but they can have their issues with food addressed and they can have their issues with anxiety or depression or manual tasks with the OT and the psychologist.(AH6 Round 1)  *someone rang me yesterday and said they're going to come out and offer me support. And then they're going to have someone come out to me weekly. And I think he said talk psychologically. Because I find going out of the house traumatic and I don't care if you come here but I don't want to come to you (PC8 Round 2)  * he [GP] has a nurse come in to do his blood tests on one and a half days a week. Then he has a podiatrist come in once a month (PC3 Round 2)  *if they were accessed by our service they would see that the diet educators and doctors all at once – so you can package the service into a one hour, two hour period, rather than, say, an admission or have a patient come back three times to see different parties (HS4 Round 1) |

| ***Valuing of WSICP***  *Participants from all stakeholder groups valued the WSICP and considered it worthwhile. They spoke about many areas:*   - *Patient self-management* - *Moving care into the community and improving relationships between GPs and Hospital* - *Change observed even in short space of time* - *Longer term benefit* - *Connecting disjointed care for chronic conditions* - *Patients and carers valuing* - *Rapid access to care* - *Improved patient quality of life* - *Patient confidence improves* - *Health care providers able to provide better service* - *Linking up HCPs to provide better care* - *Continuity and familiarity with care providers especially CFs* - *Improved patient access to providers* - *Holistic care* - *Joining up services beyond WSICP* - *Investment worthwhile* - *Time effective for GPs* - *Good return on investment with HCPs providing better service* - *Reduced admissions* - *Increased patient and provider awareness*   *Beneficial systems level changes were noted already, as seen above, and were expected to continue and grow.* | * Look, if we can help these patients manage their health better and try to get them out into the community and get them to see a GP, and manage their health, I feel like, yes, it is worth it.(HN3 Round 1)  * I’m seeing a lot more improvements, in terms of the patient’s ability to self-manage their condition and making sure they go to their GP before it becomes worse(CF3 Round 2)  * it’s actually a really huge step forward for the LHD and the hospital system to say that the care and the management of patients with chronic and complex illness is based in general practice. That is a massive shift in attitude and understanding and to a lesser extent, behaviour. I do think that’s been achieved to varying degrees certainly, and still a long way to go in terms of the attitude of many specialists. (MG3 Round 2)  * the health system where it used to be centralised, now it’s more decentralised, more for the patient’s better care, and trying to help them out in the community and manage them in community, rather than send – bring them down to the base and doing things, which is going to cost extra for the government anyway. (CF4 Round 2)  *if we can look after patients in the home setting, or in the general practice, limiting their admission time … it will certainly be cheaper (GP8 Round 2)  * the relationships between general practice and the specialists are greatly improved and therefore I think you are able to actually manage more disease better and earlier in general practice and if we keep doing this, we’re going to bend the curve a little bit (HS8 MG8 Round 2)  *trying to bridge that barrier from between the hospital and the GP, and to move the burden of care for some of these chronic diseases back to the GP, and it’s trying to create a connection that is easier to navigate. I can see how all of those things are happening (HS3 Round 2)  * I’m familiar with a lot of the challenges and difficulties and I think even in this short space of time, I feel like we’ve improved some of those things. And you can – when you know, sometimes if you can see a tangible improvement… it makes you feel that that investment is rewarding. (HS3 Round 1)  * the fact that we’re coming into a pretty hopeless situation where they’re at rock bottom, and we seem to be improving their quality of life measures, and improving their management of their chronic disease, what I’ve seen from the small amount of data we’ve got so far just seems to be quite incredible (AH6 Round 2)  * even though it’s early stages, I think it will have a significant positive impact in the future. Yeah. I think it’s a good idea (GP4 Round 1)  * I can already see that the benefits of this service will be really fantastic, and the thing that I feel we’re doing really well is targeting those people with chronic health needs who often had disjointed care and gaps in their care. So, yeah, I found it fantastic.(AH3 Round 1)  *the results that we’re starting to see and the effects so that it has a – makes an impact, so I think it is worth the sum of money and it got a more core service for the patients (AH4 Round 2)  * With the health service, the best thing I’ve ever actually come across.(PC1 Round 1)  *when they talk, they are actually keen on attending the Integrated Care Program, because they have actually seen improvements. (HN6 Round 1)  *They [patients] really like the idea that the specialist and the GP are in the room together at the GP's practice and they tell us often that they find it difficult to get into the hospital because in most places you've got a big wait list and it's very inconvenient and it's really difficult for them. But the very fact that we're out there seeing them with their GP or they know that we're building the GP's capacity is seen in a really positive light (HS8 MG7 Round 1)  *I was just one of the patients in a block of patients that were waiting to be looked after, but when I was on the program, there was an indication that I was just being sent through to be put in a ward and being X-rayed and being attended to in very quick time - very good care (PC9 Round 1)  *we’ve been able able to get people into pulmonary rehab within a month of hospitalisation and there's good evidence that that prevents rehospitalisation. So that’s been really nice, that’s worked really well. (HS2 MG9 Round 1)  *we can get these patients in, we get them seen reasonably quickly and from the point of view of the community they’re seeing an actual consultant in a couple of days. Now where can that happen in any other service? (HN2 Round 1)  * we do improve the work for the hospital in that, you know, patients are in emergency departments for less time and they are seeing their own specialists much sooner. They have a firm follow-up plan, compared to the time where there wasn’t a rapid access clinic (HS6 Round 2)  * that makes people feel comfortable when they see a face and they know they’ll see that face somewhere else and that person’s able to control their health, control their appointments, their exposure to emergency departments or try to limit that, it really did make them feel comfortable and the care facilitator role, I think, is excellent (PN3 Round 1)  *things that have been of particular value have been giving that patient the ability to have contact people in the program……the heart failure nurses that are really contactable, that gives that patient extra… reassurance, and autonomy (GP6 Round 1)  * it gives us an opportunity to look at the patient as a whole and treat them a bit more holistically, instead of just addressing their one problem at a time when they come in, which we often do in general practice. (GP12 Round 2)  *The organising of more services for the patients and having someone to help streamline that process has been really valuable as well. We’ve had patients that have needed the assistance from the Health One team as well, so that the integrated care coordinators have been able to bring that in ..so it's all … streamlined in kind of one place (GP6 Round 2)  *I think it’s – philosophy is very good. I think if it works it works. If it works it will make a huge difference and it’s certainly worth the investment I think (HS10 Round 1)  * Initially I thought it was going to create a lot more problems, like take a lot more time. If anything it's actually made it work a lot more efficiently. And care plans are now up to date. Care plans are being followed up properly - I'm definitely seeing that it's helping and I'm getting results (GP9 Round 2)  *we're more able to treat the underlying condition more effectively; we're able to address complications and then hopefully improve their quality of life. Definitely I think that's money and time well spent. I think it's a good program that is actually going to be effective (GP6 Round 1)  *ED avoidance or getting them to get out of ED quickly so they don’t need to stay in hospital for long; they can be at home and they can be linked up to come and see us the next day in the hospital. So these are all benefits that have come about. I think the value of the service cannot be underestimated in preventing admissions and improving patient outcomes.(HS11 Round 1)  *a patient who used to come in once every month, now haven’t seen him for a few months in the hospital because he’s been managed through integrated care service (HS7 Round 1)  *when I went in there she took all the usual things that they take…your weight and all that… she saw that phlegm that came out which was pretty yellow, and I told her that I’d already been on these tablets. Anyhow, she called the doctor in that works with her, and the doctor prescribed me these other tablets, which I got. And they fixed me up within a week. I didn’t have to go to the hospital (PC11 Round 1)  * it has improved because it’s not only the case of his diabetic condition but the general outgoing life, especially for the aged, for our age, it helps because I can ask some questions and the care plan giver - it’s open, so you’re not put away or whatever, so they are open to, like I would like to explain this and then they do, and it is helpful. (PC18 Round 2)  *raising awareness and having a team to deal with that and to educate emergency nurses, doctors, look at how these patients are managed in hospital; all of those things have really come about as a result of the program.(HS11 Round 1) |
| --- | --- |
| ***Suggestions for the future incl new GP models e.g. capitation***   - *Include nursing homes in Integrated care* - *Increase networking across hospitals* - *Enhanced role for allied health professionals in follow up of high risk patients* - *Vision of expanding the model to other areas of chronic illness including psychiatry* - *Review GP payment models* - *Training in IT* - *Review communication processes between hospitals and GPs* - *Explore options for client education groups* - *Expand cardiac services in the WSICP* - *Greater general practice staffing support* - *Improved IT support including easier to use* - *Changes to care plans need to be flagged* - *Ongoing investment in integrated care* - *Access to outreach for patients/carers* - *Greater engagement with non-medical community services* - *Expand case conferencing to other disciplines* - *Develop electronic capacity for case conferencing* - *Enhance community valuing of continuity of primary care and awareness* - *Review exclusionary criteria* | * Why can’t we go to the nursing homes and try and educate the nursing staff? If somebody is short of breath, rather than sending them into the hospital, they actually have qualified personnel there that can try and manage these things. But they just made that as one of the exclusion criteria.(HN3 Round 1)  * I think, we just probably need to - that networking a little bit more just to see how we’re all doing, how we’re all going, that we can, sort of, trouble shoot as well, what works for us might not work for Blacktown, but vice versa, so it would just be good(HN1 Round 1)  *all three specialities should be at the same place. Maybe, we do get these executive board meetings where we all meet up, and we look at all the data. But maybe once every couple of months, just the medical team, and the nursing team, and the allied health get together and maybe have a discussion as to how we could try collaborate and improve the system (HN3 Round 2)  * I’ve heard a number of other clinicians say, maybe we should do an integrated care model, you know, maybe we should utilise those things in this problem, so, I think that’s been interesting hearing people say we should apply similar things to other health conditions, or ways managing conditions, so, that’s exciting to me. I think that, yeah, it will work for that as well. (HS1 MG7 Round 2)  * You know one thing I’d like to add. If we could have an exercise physiologist, that would be good.(AH1 Round 1)  * if there was a community educator – diabetes educator that would be great. I think so that all the community nurses would upskill their skill as well as connect with the GP (AH1 round 1)  *people are very anxious and very depressed, and that for them is a major problem, and until you can break that barrier – so the other frustration is that we don’t have our own team down there. I have an educator with me, I don’t have a dietitian, and I don’t have psychologists. (HS5 Round 2)  * what else would be helpful would be if there was something similar for mental health where there was, like a link-up with a hospital or a psychologist or a psychiatrist, who would be able to review the patient on as needs basis, but having that would be accessible for the patients as well as (GP4 Round 2)  *our patients, the patients who are in the Integrated Care Program need more facilities, like more physiotherapy for COPD patients, they are acute and chronically sick, but only five visits they can see with the therapist (GP11 Round 2)  *a clinical psychologist, I really think there's a role for them - I've done my statistics as well and in my clinic I would say about 50% of my patients suffer from some form of mental illness, whether it’s mild or severe.(HS7 Round 2)  * They're looking at capitation funding plus pay for performance, whilst you're leaving a fee for service in place for the more episodic and acute ones. There's no way they will - the political end of the spectrum will move that far, that fast. But they may well have to move that far in the longer term. Despite the small volume of incentives that we’ve managed to bring in with this program, the system is still geared to reward high throughput, but not high value. (MG3 Round 2)  *the concept [PCMH] is good however I’m not too sure how things will work in terms of – the funding is a big issue for that and in terms of how things would work in relation to Medicare. (GP4 Round 1)  *[Health Care Homes involve] bundled payments with tiny amounts of money, and only for your sickest patients. We worked out we would be far, far, far worse off. (GP7 Round 2)  *we’re now working on E-referrals and sending out the information through the IT systems, so we could do with some more training from that side of things (HS5 Round 1)  *would be good if we could e-refer but that isn’t happening I need to fax information through (GP11 Round 2)  *we should have actually like a pathways, like a flow chart of where we can actually say where to start or where to end or where to stop. (CF4 Round 2)  * I feel a quick phone call is more useful for your GP than the long letter, and it’s persuaded me that maybe I should do a quick phone call more often…shorter, sharper communication with the GPs might be helpful, and also just understanding that we come from a completely different place (HS2 MG9 Round 1)  *what I would love to do is have a program whereby we could just send out emails, we could just send out text messages to landlines (HN5 Round 1)  *If letters were dictated and transcribed quickly then that could work. I’d probably spend an extra 10 minutes with each patient writing a letter myself. I don’t mind when we have time, but on a busy day it slows things down. (HS4 Round 1)  * we might have 12 patients booked in, they all get seen by the three doctors; there’s only one dietician. Something I have been thinking about is doing a group (AH2 Round 1)  *Something to consider in the future - in like a group setting because I think that it may be helpful for patients to see that, for example, their diabetes or COPD or whatever it is, that it’s not just them, that there are other people like them. (GP4 Round 1)  *I would say more awareness for patients – so it can be like some health education to them, I don’t know how we can go about it though. There are forums and all, but the patient’s participation might be a little bit low (GP10 Round 2)  Education of the patient and of the community as a whole, and more exercise facilities (GP8 Round 2)  *it would be good to expand it a bit, it would be probably good to look – well, we’re doing chest pain and heart failure but to link in a hypertension clinic (HN2 Round 1)  *if the GP’s unsure whether this is an acute coronary syndrome or not, then we have no choice but to refer them to the emergency department, to exclude that. I think what would make a huge difference, if we were to have the resources, then if we could function as a mini clinic that is able to exclude acute coronary syndrome, then I think GPs would feel more comfortable sending the patients our way (HS6 Round 2)  * I think it needs a little bit more investment in general practice… we probably don’t have the manpower or the staffing or the funding to employ someone to be able to track these patients and recall them in, get them in… (GP3 Round 1)  * you put a summary in shared care plan then you have seen them again and you have added all the diagnoses and you have put that back in a new shared health summary, but then you can't reimport that into the care plan (GP6 Round 2)  *find a system that would interface with Cerner so that Cerner doesn’t have to do it all. It can become the repository of it but you have intermediary systems that are much more flexible and built for purpose and let the programs talk to each other. (HS8 MG7 Round 1)  *We needed to employ some IT people in integrated care not defer to the LHD IT. (HN5 Round 2)  *if the IT was working it would make it much easier for everyone to not have any paper based communication. (HN4 Round 2)  * more allied health follow-up, so however it would be funded, allied health could take on more of a role as well, not just in sessions that they are being provided through a GP managed care plan to use a team care arrangement, but if there is some other diversion or funding so that they can then put those high risk patients on a follow-up list or make sure that they are being phone followed-up (GP6 Round 1)  *The thing that would make a really big difference would be if we could look at their notes, and they could look, maybe not everything, but if I could actually look and see what's happened. If we had a shared electronic record. (GP7 Round 2)  * my suggestion is if ever, this would be occasionally, just making an appointment or just visiting the patient at home (PC18 Round 2)  * we still don’t have a definite room or a clinic to base integrated care on. (HN6 Round 1)  * So I think a significant degree of investment in time and resources are needed to tackle that problem. It’s not going to be a quick fix problem with low budget, so yes, I think investment in resources in terms of health professionals, location and equipment and administrative support is needed (HS11 Round 1)  * tapping into that particular service with Blacktown Council. Or, in other cases, maybe a cab charge voucher that facilitates them getting to their appointment on time (HN6 Round 1)  * *diabetes do case conferencing, but how practical it would be from a cardiology point of view and how beneficial, but something where all the parties involved could get together would be good, that sort of thing, yeah (HN4 Round 1)  *I keep saying to her, I think you should do the case conferences for COPD as well. (GP5 Round 2)  *the practice can manage those cases without the specialist coming out. The [GP] would say that once that relationship is established, more of that can be done by video conference (MG3 Round 2)  *I think having the community recognize the value of having one practice providing most of their care, coordinating it, would be, on top community education would be really useful (GP7 Round 1)  *There’s not a lot of advertisement regarding the program, what we can do. I think you need a full project manager to help with communication, newsletters, you know, establishing who we are (CF2 Round 2)  *Well I would say that for diabetes, there should not be any exclusion criteria. Because people who have got complex medical problems, they need the integrated care more. (GP13 Round 2) |
